# The temporal evolution of income polarization in Canada’s largest CMAs

**DOI:** 10.1371/journal.pone.0251430

**Published:** 2021-06-08

**Authors:** Lazar Ilic, M. Sawada

**Affiliations:** Laboratory for Applied Geomatics and GIS Science (LAGGISS), Department of Geography, Environment and Geomatics, University of Ottawa, Ottawa, Canada; University of Lausanne, SWITZERLAND

## Abstract

Income polarization is a pressing issue which is increasingly discussed by academics and policymakers. The present research examines income polarization in Canada’s eight largest Census Metropolitan Areas (CMAs) using data at the census-tract (CT) level between 1971 and 2016. Generally, there are significant decreasing trends in the middle-income population with simultaneously increasing trends in low-income groups. The high-income groups have been relatively stable with fewer significant increasing population trends. Using conventional mapping and cartograms, patterns of the spatial evolution of income inequality are illustrated. Every CMA examined contains an increasing trend of spatial fragmentation at the patch level within each CMA’s landscape mosaic. The results of a spatial autocorrelation analysis at the sub-patch, CT level, exhibit significant spatial clustering of high-income CTs as one process that dominates the increasingly fragmented landscape mosaic.

## Introduction

Inequality is an increasingly pressing issue. As a popular element of social justice [1], inequality is exacerbated through uneven development at a multitude of spatial scales, exposing the contrasts between haves and have-nots. Whereas some studies concerning inequality focus on an international or national scale [2], recent research increasingly includes local income polarization at regional and urban scales [3–8]. Income polarization is a process where the middle-income group decreases in size while the two opposing poles of low-income and high-income groups expands in size.

In the Canadian context, a network of researchers who are part of the Neighbourhood Change Research Project conducted a number of studies on income inequality in select Canadian cities, the results of which are summarized in a recent publication [9]. Herein, we refer to, their methodological approach as the Three City Project (TCP), due to their utilization of what has come to be known as the Three City Model (TCM).

We posit that research on income polarization requires an examination of time-slices in order to determine how the spatial pattern of income groups change over time. A time-slice map portrays the spatial pattern of the income groups (low, middle, high) for a particular census year. Time-slice maps of income structure build on work by MacLachlan and Sawada [10], who identified an income range as a methodological necessity for examining income polarization.

This present research addresses income polarization, while focusing on the following questions: Given that previous research suggests that income polarization has increased in certain Census Metropolitan Areas (CMAs) over the past few decades [9–12], how has income polarization evolved in Canada’s eight largest CMAs between 1971 and 2016? To what extent are low-income and high-income groups changing at the expense of the middle-income group? How is income inequality manifested spatially within CMAs? For example, are income groups more fragmented within the CMAs and, if so, is there spatial clustering of income groups?

## Justifying income as a measurement of inequality

Inequality is manifested in numerous ways, including but not limited to happiness, wealth, opportunities, achievements, needs, freedoms, rights, quality of life, and so forth [13]. For the purposes of this study, the type of inequality being examined is income inequality.

There are numerous multivariate indices of inequality and several inequality indices include, income in addition to various socioeconomic variables [14–18]. Furthermore, income is a dominant variable in measures of socio-economic status (SES) [19, 20].

Justifying income as a sole measure of inequality is usually glossed over in most income-only studies, due to an implicit assumption that income is a valid *a priori* means of measuring inequality. Axioms postulated include: that income is the “most common and encompassing measure” for examining inequality [21], that “income is the key contributor to the well-being of Canadian families” [22], and that income is the “most used indicator” for examining economic inequality and segregation [4]. However, such statements regarding income are equivocal justifications at best. Thus, *a priori* claims regarding income as the most common or best measure of inequality are debatable.

Critics of income-only inequality suggest that income does not capture the extent of opportunities that people face [13]. The use of only income may be problematic because it neglects deprivations which relate to other socioeconomic indicators such as unemployment, health, education and social exclusion [23]. These variables however, particularly health and social exclusion, can be hard to quantify and obtain in the Canadian context.

Income is a powerful variable because it is scalar, readily available, and amenable to statistical analyses. The basic categorization of needs for which income is a prerequisite is depicted in Maslow’s hierarchy [24], which compartmentalizes human needs into five categories. In the context of Western industrialized countries, particularly in urban areas, income is a prerequisite for the fulfilment of the most basic needs such as food and shelter. If an individual has no income, then they would fail at realizing any of the needs in Maslow’s hierarchy. Furthermore, individuals with larger incomes have more freedom to choose how to utilize their income to best satisfy higher-level needs. Thus, while income does not capture other aspects of deprivation, generally speaking, income is a prerequisite for a number of other dimensions such as those noted by Sen [13], including health, education and the satisfaction of basic needs.

Consequently, several scholars have chosen to focus on income as a stand-alone measure of inequality. Family well-being implicitly implies financial security which is realized through income. This is not to say that income is the ideal variable in every case. Whereas Esteban and Ray [25] claim that income is a good proxy for socioeconomic differences or similarities, others such as Walks [26] prefer to focus on wealth because they feel that it better captures class difference. However, obtaining wealth data is more arduous than obtaining data on income, which is the simplest and most general indicator of welfare and inequality [27].

On a final note, in recent decades, the implementation of neoliberal policies by municipal governments, through the movement from managerial to entrepreneurial means of governance, has produced a scramble to recoup revenue via imposed or increased costs using pay-for-service charges [28–32]. In such context, income has become increasingly important in one’s ability to maintain the same life choices and activities, be they education, access to recreational spaces, or the handling of added costs for utilities and infrastructure usage. The importance of income to deprivation in general is underlined by basic income projects that are increasingly discussed and attempted in affluent countries [33–36]. Such projects would in theory ensure a minimal income through which individuals can meet their basic needs.

### Inequality vs polarization

This paper makes use of two terms which are similar but slightly different: income inequality and income polarization. Income inequality refers to an unequal distribution of income [37], and, as such, can be manifested in different ways [38]. Income inequality can exist without income polarization. Conversely, income polarization is a term that is focused on the disappearing middle-income segment of society [39]. Under income polarization, middle-income groups decrease while low- and high-income groups increase [10, 40, 41].

Income polarization is a type of vertical inequality. Vertical inequalities are inequalities in terms of income between either households or individuals [42]. Other inequalities exist too. For example, horizontal inequalities are between socially constructed groups, and as such are with respect with categories including but not limited to gender, ethnicity, sexuality, and so forth [43]. Horizontal inequalities are also those that are spatial in nature. We are interested in exploring how vertical income inequality plays out spatially, for example, to what extent is the income group mosaic changing with changing income inequality in Canadian CMAs.

### The Canadian context

The concept of “polarization” was popularized by Harvard’s economist Lawrence Katz in the mid-2000s [44]. It is under that backdrop, and subsequent interest in inequality after the economic crisis of 2007–08, that recent studies of income polarization in the Canadian urban context commenced [5, 11]. Income inequality in Canada is quintessentially an urban phenomenon [45]. Considering that Canada is one of the most urbanized countries of the world (with about four fifths of its population residing in urban areas [46, 47]), urban issues are national issues. Research since the 1990s shows that income inequality has increased in Canada (on national, provincial and urban scales), to the extent that the country experienced the highest rise in inequality amongst OECD countries since the mid-1990s [3, 45, 48–52]. Such trajectories in income composition and distribution are of concern, because governments will at some point have to seriously address this development in order to evade adverse ramifications such as economic and social instability [53].

## Geographic approaches to measuring income polarization at the census tract level in the Canadian urban context

In the early 1990s, urban geography research examining divisions within cities flourished [54–59]. Concomitantly, studies on income inequality and polarization in the Canadian context also emerged [10, 52, 60]. MacLachlan and Sawada [10] detected income polarization by examining how the middle-income group changed in twenty-two Canadian CMAs. Their key contribution was the delineation of income ranges (e.g., low, middle, high) for examining income polarization. This is important, because a city’s income distribution may both polarize and depolarize depending on how the middle is defined, and that therefore it is necessary to examine different ranges in order to detect if polarization is actually occurring [10].

About a decade later, research on income polarization was picked up by the Three City Project (TCP). The TCP originated as a report that compared trends in income change of census tracts (CTs) in Toronto solely between two endpoints: the 1971 and 2001 censuses [5]. When the second report [11] was published, the production value increased in terms of improved visuals. The second report resulted in TCP research gaining more traction and notoriety (S1 in S1 Appendix). In this updated review, maps for additional census years were produced which demonstrated a more detailed evolving income landscape in Toronto between 1971 and 2006. Thus, emerging concentrations of high-income and low-income areas were illustrated.

After the updated report, numerous studies ensued which examined income polarization in other Canadian urban areas [12, 61–64]. Unfortunately, the TCP research across select urban areas did not use the same analytical parameters. These differences limit ability to make broad generalizations between cities.

In effect, the TCP produced two products. The first is the “Three City Model”, which the researchers present as an examination of income polarization. However, that model actually examines income trajectories [65].

The second TCP product examined income-polarization through “time-slices”. These time-slices cartographically convey, in a cartographic manner, what previous studies [10] discussed. However, the TCP researchers did not make maps for every census year, nor did they examine the spatial patterns of income inequality across urban areas. Spatial information can support more robust conclusions regarding potential knock-on effects of spatial inequalities to, for example, urban form and function.

## Methodology

This research examines the temporal evolution of income polarization. We begin by investigating how various income groups have changed over time in each Census Metropolitan Area (CMA). Analyzing the trend of each income group over time allows for direct comparisons of the slopes of the trend lines that model the rates of income decline or rise among the CMAs in our study. Subsequently, an examination of the spatial distribution of income groups within each CMA is achieved using time-slice maps. Finally, time-slice maps are subjected to spatial analysis to measures fragmentation and spatial autocorrelation within CMAs.

### Study areas

**Fig 1:**
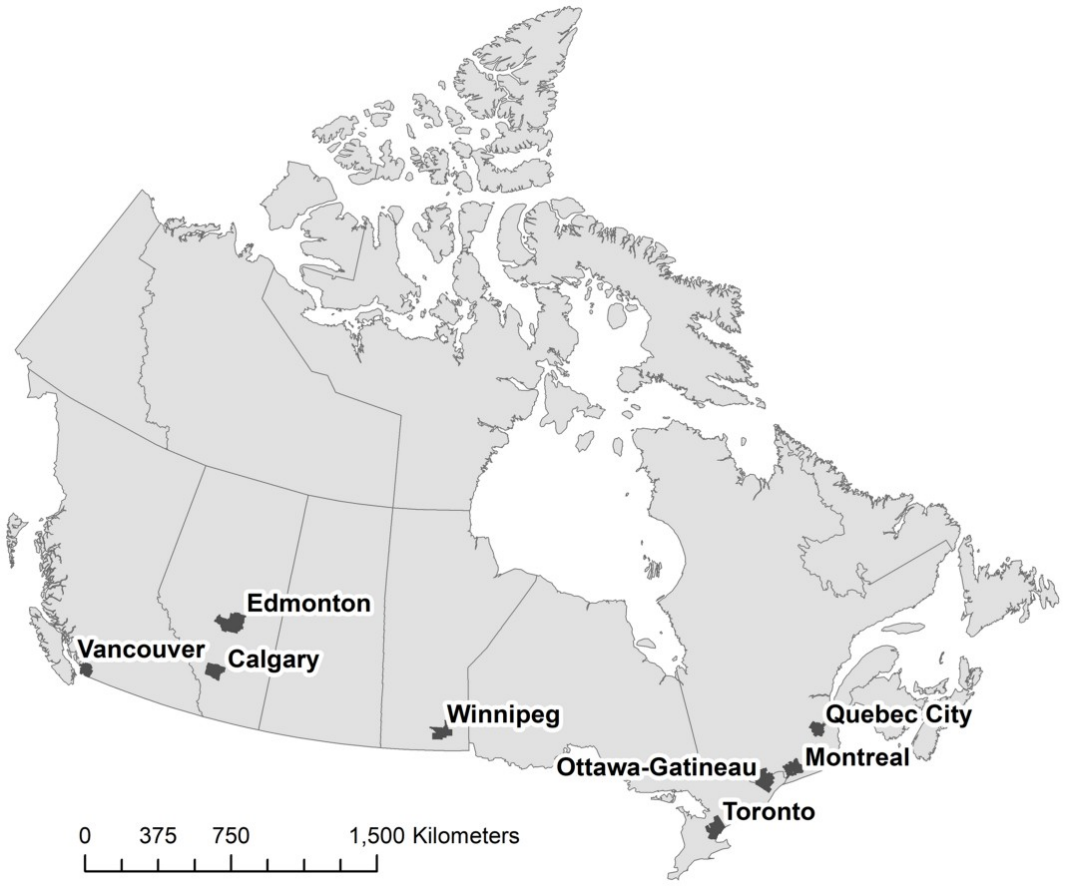
Locations of Canada’s eight largest CMAs.

This study examines the eight largest Census Metropolitan Areas (CMAs) in Canada: Toronto, Montreal, Vancouver, Calgary, Ottawa-Gatineau, Edmonton, Quebec City and Winnipeg (Fig 1).

In this study, the spatial boundaries used are the CMAs of each census year. The CMA is a popular geographic level of analysis, and can be rationalized by the notion that the CMA reflects the labor market [11].

For this study, within each CMA, analysis is at the census tract (CT) scale, which is the most common spatial unit at which cities and urban processes are examined (S3 in S1 Appendix). Data at this level of geography is available throughout all years of study.

### Data

The data for this study is from Statistics Canada and covers eight census years between 1971 and 2016. The 1976 census is considered to be a mini census, and it was only starting in 1986 that mid-decade censuses in Canada collect the same items as the start of decade censuses [66]. We do not use 2011 census data because politically motivated issues led to a non-random population sample on much data. The federal regime at the time made the long form census voluntary, resulting in a disconcerting situation whereby data scientists were effectively “muzzled” by not having access to quality data [67].

The *focal variable* for this research is average before tax household income at the CT level. In the present paper we usually refer to this census variable as simply income. The full census variable names used are provided in the supplemental material (S4) in S1 Appendix. Previous research used income at the household level [10], but without presenting a very strong rationale for the choice. Households may represent atomic units of the urban landscape more so than individuals, as individuals pool their assets towards major expenditures. The largest such cost remains the same throughout decades: housing / shelter costs [10, 11]. Using average household income in lieu of average individual income should yield similar results [11]. Finally, out of convenience it is easier to use average household income because the census provides the former in all census years that we examine. However, to assess the robustness of our results under different assumptions, we repeated our income trend analysis using the aforementioned methodology on individual-based income.

The missing data for 1976 and 2011 was imputed using linear interpolation. Moritz et al [68] tested the accuracy of numerous imputation techniques on time-series which had various levels of periodicity, trend, random and non-random missing data. They found that linear interpolation was the most accurate means of imputation for non-periodic datasets with a trend and missing data that is assumed random in nature. While our time-series of high-, middle- and low-income is quite short (ten census years), it exhibits no obvious seasonality but does show trends. As such we choose to use linear interpolation to impute the values for 1976 and 2011 prior to statistical analyses.

We use income figures before taxation, because after-tax income data was not available until the 2006 census [69], and median income was not collected every year. Hence, to maintain consistency for all years, we opt for the common pre-tax average for all analyses. In the literature there is no consensus as to the ideal measure of income that should be used when measuring income polarization [5, 10]. Thus, we feel confident in using before-tax income through time to assess the temporal evolution of income polarization.

The total number of households was not available in the 1971 census. However, numbers of households within income ranges were provided. Hence, a sum of the households in all the ranges gives a number sufficiently close to the total number of households, varying only due to rounding issues (because Statistics Canada often rounds figures to the nearest number ending in 5 or 0).

The average income of households in the CMA was not available for the 1971 census. This is the only year in our study which has such an anomaly and hence the average income figure had to be derived using the total number of households with income, and the average household income in each CT. The formula for the CMA’s average household income is:

$$AI_{c}=\sum_{k=0}^{n}{\;(\frac{HH\times {AI}_{hht}}{T_{HH}})}_{k}$$

Where AI_c_ = Average Income of CMA, where n = total number of CTs, AI_hht_ = Average Household Income of CT, HH = Households with income in CT, T_HH_ = Total households in study area.

This method was validated on data sets from years where average household income was provided for the entire CMA. For census years in which Statistics Canada provides average income figures, our calculated averages were within a fraction of a percent of the provided figures.

All data that was used in this study has been made available on the following Github page: https://github.com/lazarification/IncompePolarization_CA_CMAs.

### Population based weights

Previous research has used population weighted income for census units when examining inequality in North American cities [53, 70, 71]. Such methodological decision is in lieu of treating all CTs in an area as having equal weights of one.

Assigning household weights is rationalized through the notion that CTs with higher population (i.e., households) have more impact on their corresponding income group (low, medium and high) than CTs with lower population. Such an approach is similar to the work of MacLachlan and Sawada [10], who calculated the sizes of middle-income groups, in various CMAs at the CT level, based on the total number of households in CTs whose average household incomes corresponded to such income group.

In our analysis we employ household weights when assessing trends of low-, middle- and high-income groups, as well as in the production of cartograms. We do not employ these weights to produce conventional maps, preform fragmentation indices, or to conduct spatial autocorrelation since these latter analyses do not consider a continuous variable.

### Defining and measuring the middle

The middle-income group is most often defined by using an income range, and these *vary across studies*. For example, in the Three City Project [5, 11, 12, 61–64], the range used was ±20 percent around the average income. This 40-percentage point range, hereafter referred to as the bandwidth, is between 80 and 120 percent of the average income. However, numerous other definitions of the middle exist. Incomes which range from -33 percent and +100 percent of the median income are considered middle-income by Kochhar et al [72]. Piketty [73] defined the middle as the 40 percent of the population which is above the median and bellow the elite (who he classified as top 10 percent).

Interpretations are less robust in the absence of any means to invalidate observed trends that emerge from a single arbitrarily middle-income bandwidth definition. Previous research has shown that, depending on the chosen bandwidth, results could be engineered to exhibit either polarization and depolarization [10]. It is, therefore, important to assess the sensitivity of results, to broadly different bandwidth definitions.

Sensitivity analysis examines multiple bandwidth definitions for the middle-income group to determine the consistency of income trends in each bandwidth between 1971 and 2016. TCP researchers did not examine multiple definitions of middle-income ranges when they analyzed time-slices in Canadian cities. Without a common definition of ‘middle’, the comparison of trends between different CMAs is fraught with potentially spurious inferences.

This study uses **±**15, **±**20 and **±**25 percent bandwidths to assess the sensitivity of the low-, middle- and high- income trends and also presents the average of the three as a robust trend. The mean of the three bandwidths for each time-slice provides results that are less sensitive to any single arbitrary change in magnitude induced by an income group’s bandwidth for a particular year. The average ensures that any outliers, in the form of a high-frequency variation within one of our three bandwidths is reduced in each year. We use the non-parametric mean for each time-slice and recognize that the assumptions of normality for a given year are not valid and so use Monte-Carlo methods to assess the significance of trends in our data.

Once CTs are assigned a corresponding income range, it is possible to sum their household counts to produce a total household weight in the form of a percentage of the total CMA households. This is also done for the low- and high-income categories, because one should identify if these other two groups both increase in order to be able to affirmatively deduce that income polarization is occurring [74].

To determine how low-, middle- and high-income groups have changed over time, graphs are produced which plot the percentage of each income group over the temporal duration of the censuses examined. From each graph, we modeled the linear trend of the mean of the three bandwidths and derived a slope value to assess if the particular CMA exhibited rise or decline in each income group over time.

To assess the statistical significance of the linear trend lines from 1971–2016, we obtained confidence intervals for the mean slopes for each income group for each CMA using non-parametric bootstrapping, replicating 10,000 bootstrapped slopes for each income group’s slope by randomly sampling, with replacement.

### Mapping

We utilized a ±20 percent bandwidth in creating our maps. We map every census year in which data is available and produce cartograms, weighted by the number of households in order to provide a more balanced representation of the reality of income polarization in urban areas.

Examining the spatial distribution of income polarization within the boundary of CMAs, at the CT level, provides excessive visual weight for rural CTs that are large in area but low in number of households. Large-area CTs obscure the significance of small-area CTs within the more populated city centers, hence the use of cartograms. Cartograms weight the CTs by the number of households, thereby decreasing the size of CTs with smaller numbers of households and increasing the size of CTs with larger numbers of households that fall into a specific income category. As such, small-area CTs with high numbers of households are visually expanded in area on the map. The goal of the cartogram is to produce a topologically enforced density equalized map of a CMA. Density is equalized by changing the size of census tracts.

Cartogram production used the Gastner-Newman algorithm [75]. Cartograms by definition yield twisted and distorted images [76]. Contiguous cartograms require multiple iterations in order for the distorted CT shapes to become progressively closer to matching the chosen weight [76], which in our case is the total number of households per CT. Therefore, we applied four or five iterations of the Gastner-Newman technique for producing each cartogram. This amount of iterations was chosen because the application of additional iterations produced images which appeared almost identical, meaning that there was a convergence of the Gastner-Newman algorithm and only minor changes in density, whereby the size of modified CTs corresponds approximately to the total household weights. Certain CT boundaries had to be corrected in certain CMAs for select years due to mistakes in source digitization (S5 in S1 Appendix).

### Fragmentation analysis

As the inequality of an urban area changes, we expect concomitant changes in the spatial distribution of the three income groups as a consequent of numerous urban processes related to income polarization. For example, gentrification, among others, leads to changes in the income status of adjacent census tracts, due to, for example, spillover effects [77]. As such, fragmentation does not show income inequality itself, rather it reveals the spatial structure that can stem from income inequality. Hence, while fragmentation is not per se income-polarization, we are using it as an additional tool to examine the consequences of income-polarization spatially which can provide insight on whether the income trends lead to a more or less divided urban landscape.

From a municipal perspective and under the purview of social justice, understanding socio-economic fragmentation of income groups within CMA boundaries can aid in developing urban strategies aimed to decrease spatial inequalities in access to services, health and so forth. Additionally, the knowledge of the spatial configuration of vulnerable populations can aid in developing strategies to mitigate undesirable socioeconomic conditions by better targeting places and populations in need.

Patch fragmentation studies usually involved examination of landscapes in an ecological context [78–84]. Likewise, the urban spatial structure is characterized as fragmented [85]. Consequently, fragmentation studies have been applied to demographic data [86], and more recently in urban contexts [87–89].

Herein, the socio-economic fragmentation of each time-slice map is calculated. A fragment or patch is defined as any contiguous set of CTs that belong to the same income category (low, middle or high). These groups of CTs create a mosaiced landscape composed of high-, middle- and low-income patches. CTs with data suppression are not considered in the analysis. Water bodies are present in many CMAs, and in effect form lacunas of empty space that divide CTs. Major water bodies (large rivers, bay inlets, oceans, etc) were present in some CMAs and were treated as dividing lines that separate fragments/patches. Thus, groups of contiguous CTs were split. However, we did not split individual CTs which were multipart polygons.

As water bodies are not present in all CMAs, it was necessary to remove them from certain digital boundary files.

Two patch fragmentation indexes were used: the Johnsson Fragmentation Index and Edge Density. The lower limits of both of these indexes approach zero in situations when the total area examined is large and there are either few fragments or when there is relatively small fragment edge length. Conversely, the upper limit is boundless.

Johnsson’s Fragmentation Index is given as [82]:

$$Fragmentation\;Index=\frac{\left(M-1\right)}{\left(N-1\right)}$$

where M is the total number of fragments (or map regions), and N is the total number of areal units (or pixels in the raster). For this index it is therefore necessary to obtain the total number of fragments and to convert the CTs to raster format in order to obtain the total number of pixels. The cell size for conversion was set to 50 m^2^ (only the absolute value of the index is sensitive to differing cell sizes of 10 m^2^, 100 m^2^ and 500 m^2^, but the trend invariant).

Edge Density is a more common fragmentation index and can be expressed as [86]:

$$Edge\;Density=\frac{\sum e}{a}$$

where e is the perimeter of each class of polygons, e.g., middle income, and *a* is the total area in question.

Both fragmentation methods contain area related terms in their denominators. The methods are effective when comparing similar spatial extents, but their results are respectively incomparable when the study area’s spatial extent changes drastically even though both are normalized by area. An example is provided in the supplemental material (S6) in S1 Appendix, demonstrating the necessity of calculating fragmentation in this research using a constant sized study area for each CMA.

For each city, in order to construct a constant study area for the fragmentation indices, boundary files for each CMA for all census years were spatially intersected. The resultant common area was used for fragmentation analysis. The areas outside this common boundary mainly were comprised of water bodies and sparsely populated rural areas in most cases.

Constructing a constant spatial boundary through time using geometric intersection resulted in numerous slivers or spurious polygons. Such polygons result from discrepancies in the digitization of CT boundaries in different census years—often the boundaries for the same CT do not perfectly overlap from one year to the next. These spurious polygons are a larger concern for the Johnsson Index, and hence were not used towards the total fragment count.

Using a constant study area essentially results in the Johnsson Index becoming effectively a count of Fragments divided by a constant area, while the Edge Density measure amounts to a sum of edge lengths divided by a constant area.

### Spatial autocorrelation

The fragmentation indices analyze the high-, middle- and low-income mapped CMA mosaic at the patch level for each year. A patch is composed of one or more CTs with the same income status. As such, the patch-level pattern is an emergent pattern resulting from the non-spatial process of income polarization as well as spatial organizational processes operating at the level of the CTs. As such, to complement the patch fragmentation indices, we assessed the degree of spatial autocorrelation within each of the income groups for each CMA and each time-slice. The examination of changes in the spatial autocorrelation of each income group can help us understand what spatial processes (clustering/dispersion) are giving rise to the patches and whether or not patches tend to grow or shrink over time with changes in the income trends.

A nominal level measure of spatial autocorrelation (SA) called the Joins-Count (JC) is used herein to compute spatial autocorrelation for each time-slice map [90]. Calculation of the joins-count measure utilized the ‘joinscount.mc’ function in the package spdep [91] in R 4.0 [92].

The JC enumerates the number of ‘joins’ between polygons with categorical labels. A join occurs whenever two CTs share a boundary. On a time-slice map, each CT is a polygon that is contiguous with its neighbouring polygons. For example, if two CTs are contiguous via a shared border then that border represents a single ‘join’ between the two CTs. The JC measure counts how many times a join is found between polygons that belong to the same nominal category (e.g., between two middle-income CTs) or between different nominal categories (e.g., middle and high or middle and low or high and low).

Joins counts are preformed using a binary spatial weights matrix. The analyses for each time-slice was completed using a Queen’s case neighbourhood definition, wherein a given CT included all surrounding CTs as neighbours if they share a boundary or point along their boundary. Because spatial autocorrelation results can be sensitive to the definition of neighbourhoods used, we tested the sensitivity of the results by repeating the analysis using both a Rooks (sharing only a linear boundary) and K-nearest-neighbour (with k = 5 so that each CT has 5 neighbours) neighbourhood definition.

In each map, there are three income categories (low, middle and high), and the JC was used to determine the number of low-low, middle-middle and high-high joins. If, for example, CTs for the middle-income group tend to be joined more often to other middle-income group CTs, then that income group on a time-slice map is an example significant positive spatial autocorrelation of middle-income CTs. Positive SA indicates that the income category tends to be spatially clustered rather than dispersed within the CMA.

For a given time-slice map, to estimate the expected value and the variance for each income category, 999 simulated joins-count measures were produced by shuffling all three income group labels on the map and each time counting the number of joins for each income group and adding the observed number of joins as one possible outcome. Next, the mean and variance for that simulation was calculated and stored. Then, for each map that same procedure was repeated 1000 times to create two normally distributed distributions, one containing the means and the other the variances. The average of the means and variances of the reference distribution then approximates the true expected value and variance for an infinite number of joins-counts measures for the given time-slice map. Finally, for a given map, the observed number of joins for each income category was subtracted from the average of the reference distribution and divided by the standard deviation to arrive at a standardized z-value for each income group in each time-slice in each city. Higher z-values indicate stronger positive spatial autocorrelation for a given income group whereas lower z-values near zero indicate weaker positive spatial autocorrelation and negative z-values indicate significant negative spatial autocorrelation. When assessing a given plot for significance, we only interpret the trendline if at least one of the time slices exhibits a z-value that corresponds to the Bonferroni corrected rate of α = 0.005 which corresponds to z = 2.576. The reasoning is such: significant spatial autocorrelation values across the time slices are no different from a random shuffling of the three groups among the CTs and thus a trend would be spurious.

For a given time-slice and income group, a joins-count measure that increases over time suggests a clustering process, wherein similar income groups are more often found together (by inference of sharing numerous joins), perhaps because of spatial segregation of income groups for example. Conversely, if the joins-count measure is decreasing over time then this suggests that there is a tendency for CTs with different income group memberships to be adjacent. Under a decreasing joins-count scenario, an income group would be more likely to find a different income group adjacent to itself and thus the group in question would be more dispersed than chance would predict. Such a scenario can be explained by increased fragmentation of CTs of that income group within urban landscape mosaic.

The joins-count z-values were plotted over time and the linear trend was modelled and bootstrapped to obtain confidence intervals for the 24 panels (one per three income groups in every CMA) using the same procedure as was used for the average of the three bands for income groups detailed above.

## Results

**Fig 2:**
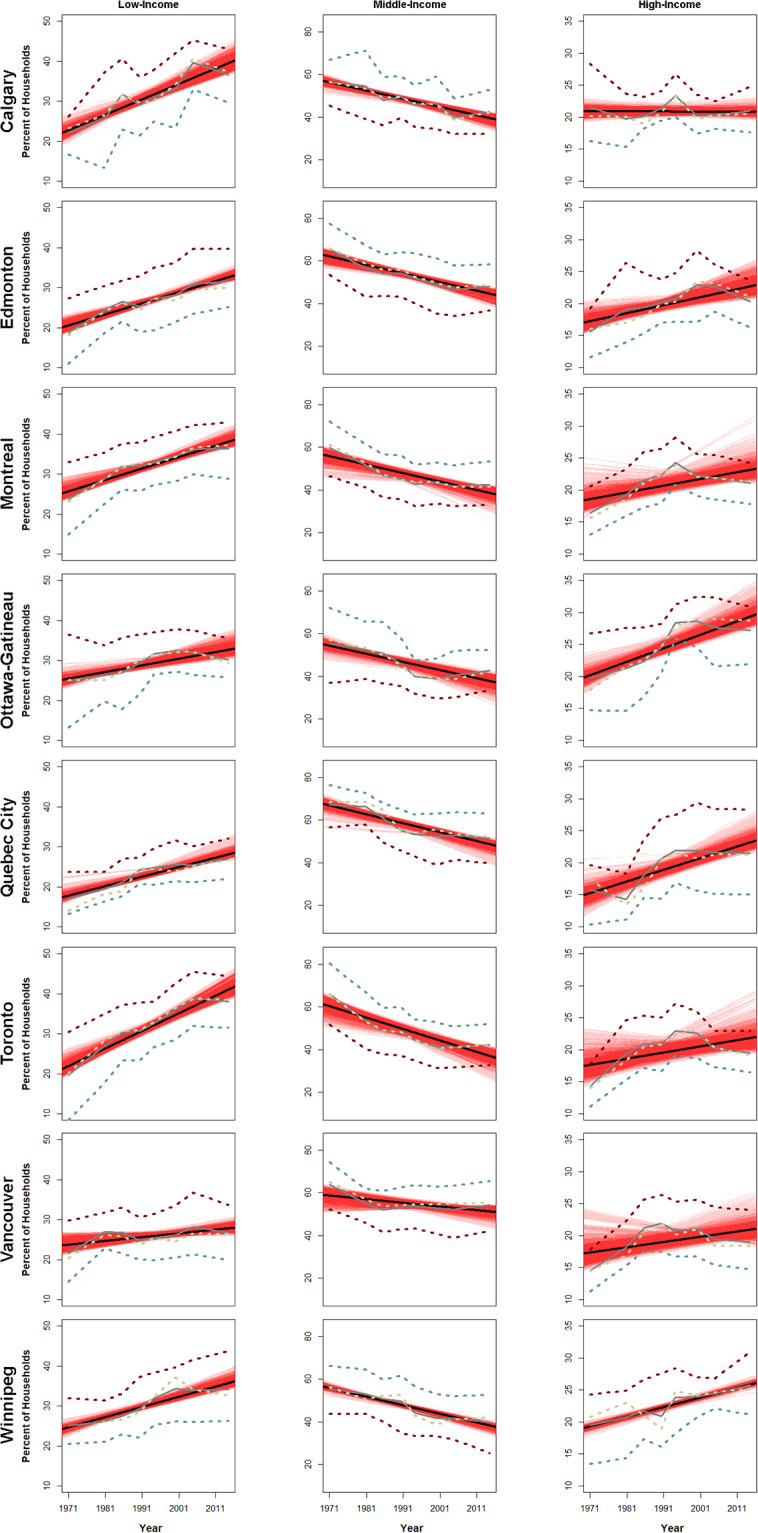
The percentage of households in low, middle and high-income classes, in Canada’s largest eight CMAs. Each column has a different y axis, but each panel within the same column has the same axis range. Different definitions of the middle are shown (blue: **±**25 percent of average, yellow: ±20 percent of average, dark red: ±15 percent of average), with grey being the composite average. Confidence intervals of the linear trend of the average of the three bandwidths are shown in red. A bootstrapped confidence interval that does not cross zero indicates a statistically significant result.

The statistically significant negative slopes for the middle-income group trend line in all cities (Fig 2) based on household (Table 1) or individual (Table 2) income data implies a steady erosion of the middle-income group since 1971 for all CMAs except for Quebec City and Vancouver. Additionally, all CMAs have significant positive trends for the low-income group under either household income (Table 1) or individual income (Table 2), with the exception of Quebec City in the latter. Given that these are proportions weighted by the number of households, the low-income group is expanding within most CMAs at the cost of the middle-income group. For household income, high-income groups exhibit increasing trends but only half are statistically significant (Table 1). Three-quarters of the high-income group trends are significant if we consider household income (Table 2). In general, middle and low-income groups show similar trends for both types of income, household or individual. The greatest variability is around the high-income group. For all subsequent analysis and mapping we utilized the average household income data only.

**Table 1:** Slopes of the three income groups (low, middle & high) and their associated 95% confidence intervals (CI) obtained via non-parametric bootstrapping of household income data.

CMA	Low (95% CI)	CI_mid_	CI_high_
Calgary	0.37 [0.27, 0.48]*	-0.37 [-0.47, -0.27]*	-0.00 [-0.03, 0.04]
Edmonton	0.27 [0.21, 0.32]*	-0.39 [-0.52, -0.26]*	0.12 [0.04, 0.21]*
Montreal	0.28 [0.19, 0.36]*	-0.38 [-0.57, -0.17]*	0.10 [-0.02, 0.21]
Ottawa-Gatineau	0.16 [0.06, 0.25]*	-0.37 [-0.55, -0.17]*	0.20 [0.12, 0.29]*
Quebec City	0.23 [0.16, 0.29]*	-0.41 [-0.54, -0.25]*	0.18 [0.10, 0.27]*
Toronto	0.42 [0.31, 0.52]*	-0.52 [-0.77, -0.29]*	0.09 [-0.03, 0.26]
Vancouver	0.09 [0.02, 0.16]*	-0.17 [-0.35, 0.01]	0.08 [-0.05, 0.21]
Winnipeg	0.25 [0.20, 0.31]*	-0.39 [-0.46, -0.34]*	0.15 [0.14, 0.16]*

A slope is significant statistically when its confidence interval does not include zero and these are indicated by an *.

**Table 2:** Slopes of the three income groups (low, middle & high) and their associated 95% confidence intervals (CI) obtained via non-parametric bootstrapping of individual income data.

CMA	Low (95% CI)	CI_mid_	CI_high_
Calgary	0.57 [0.45, 0.74]*	-0.68 [-0.86, -0.55]*	0.11 [0.08, 0.14]*
Edmonton	0.35 [0.27, 0.43]*	-0.48 [-0.60, -0.32]*	0.13 [0.05, 0.18]*
Montreal	0.34 [0.17, 0.54]*	-0.42 [-0.65, -0.24]*	0.08 [0.05, 0.11]*
Ottawa-Gatineau	0.08 [0.05, 0.15]*	-0.12 [-0.23, -0.04]*	0.05 [-0.02, 0.12]
Quebec City	0.02 [-0.02, 0.06]	-0.05 [-0.14, 0.02]	0.03 [-0.01, 0.8]
Toronto	0.53 [0.41, 0.66]*	-0.62 [-0.76, -0.48]*	0.09 [0.07, 0.13]*
Vancouver	0.11 [0.08, 0.16]*	-0.51 [-0.61, -0.43]*	0.40 [0.35, 0.48]*
Winnipeg	0.34 [0.28, 0.39]*	-0.51 [-0.58, -0.43]*	0.17 [0.11, 0.26]*

A slope is significant statistically when its confidence interval does not include zero and these are indicated by an *.

Fig 2 illustrates that, for a given bandwidth definition (e.g., ±15%, ±20%, ±25%), there is variability in the percentage of households over time within any of the three income-groups. In many cities, the census to census variability between the different definitions of the middle differ only in magnitude between the three bandwidths. For example, in several cities, one could choose any bandwidth, say ±15%, and the others (±20% or ±25%) would be a simple translation of the curve along the percentage axis. However, in other cases, the variability for a given bandwidth is less predictable for any randomly chosen census year.

In many of the middle-income graphs (Fig 2), from 2006 onwards, the bandwidth curves stop declining and at the same time many low-income group trends plateau. There are fewer significant positive trends in the high-income group across the CMAs, thus the middle-income group has not shifted by equal amounts to the low- and high-income groups. Growth of the lower-income groups over the higher group has been previously observed by Pahl [93].

**Fig 3:**
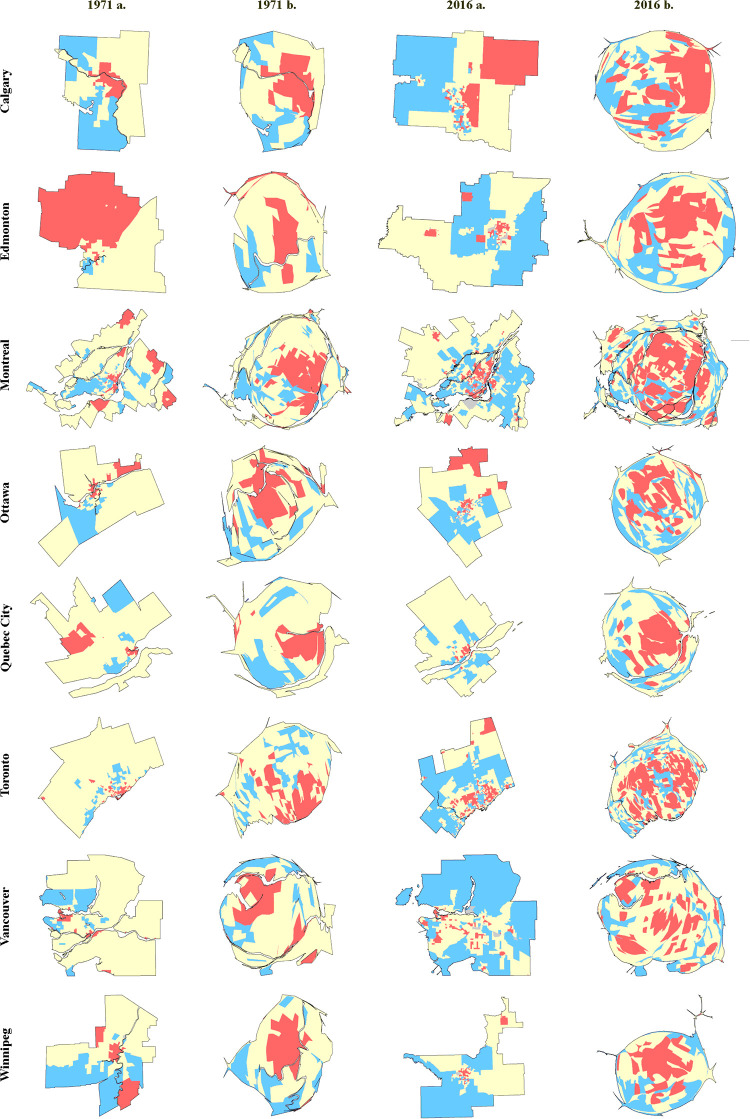
Conventional maps (a) and Cartograms (b) of the temporal evolution of the income structure of Canada’s eight largest CMAs. Low-income CTs (less than 80 percent of average) are in red, middle-income CTs (±20 percent of the average) are in yellow, high-income CTs (above 120 percent of the average) are in blue, and other (no data, or no households) CTs are in grey. Additional maps for each census year for all eight CMAs are provided in the S1 Appendix.

Maps (Fig 3) provide insight into how the income groups are spatially structured through time. For considerations of space in the main body of this paper, maps are provided for only the time-period endpoints of 1971 and 2016 for each CMA (Fig 3). However, sets of maps are provided for each CMA for every year in the supplementary material (S7) in S1 Appendix.

Conventional maps show how the CMAs have expanded (and in some cases temporarily contracted) in size over time (S6 in S1 Appendix). Between 1971 and 2016, both high- and low-income groups have expanded from the center towards outer regions of many CMAs.

CMAs include suburban and rural census tracts (CTs) which have large areas. The density equalization effect of the cartograms is effective in reducing the strong visual weight given to rural CTs with smaller numbers of households. Likewise, cartograms increase the size of CTs that are closer to the central areas of CMAs, which are territorially small but contain equal or greater numbers of households.

In every CMA, there has been a significant decline in the middle-income trend since 1971. Spatially, the gap left by the middle-income group decline is illustrated by cartograms that show expansions of low- and high-income areas (Fig 3 and S6 in S1 Appendix). Concurrently, the low-income areas (in red) have expanded considerably.

Visually, all CMAs appear more fragmented over time (Fig 3 and S6 in S1 Appendix). Both conventional maps and cartograms support this observation, as over the decades an increasing number of separate fragments have emerged in every CMA. This is perhaps most visible in Vancouver’s CMA, where one can observe a mosaic of many fragments forming rather than large clusters of CTs.

There has been an almost continual increase in fragmentation between income groups in every CMA. Some regions, such as Ottawa-Gatineau exhibit continual increases in fragmentation. Others have moments of stability before fragmentation resumes on an upward trend. Examination of the indices calculated on the constant area, Montreal, Toronto and Calgary show the steepest increases, while Winnipeg, Quebec City and Ottawa-Gatineau exhibit moderate increases and, finally, Edmonton and Vancouver show the smallest increases in fragmentation since 1971. The indices calculated on the full complement of CTs in each year tend to show very low increases in fragmentation and some decreases (Ottawa-Gatineau and Winnipeg).

**Fig 4:**
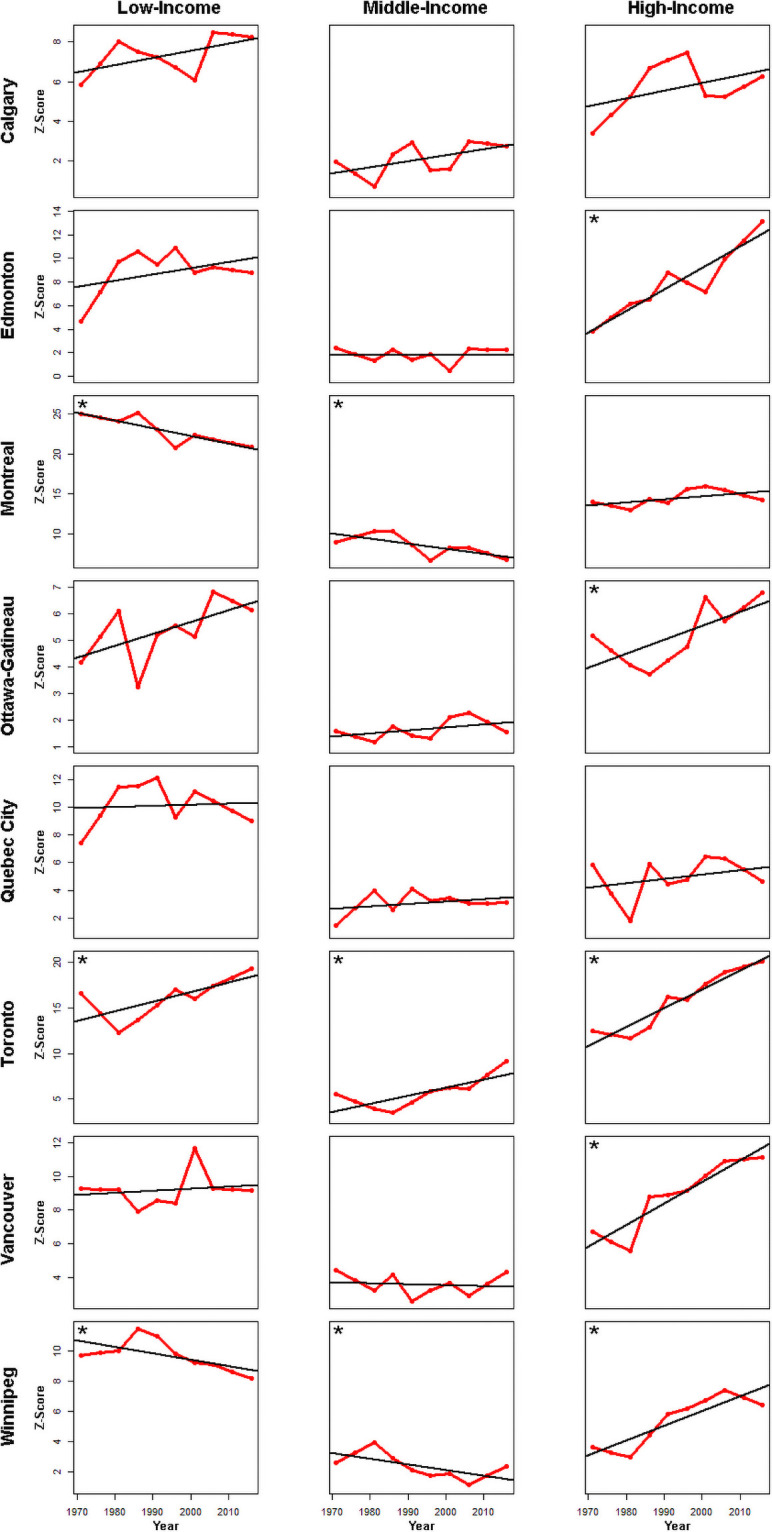
Results of spatial autocorrelation / join count analysis. Confidence intervals from bootstrapping are available in the supplementary material (S9) in S1 Appendix. Significant trends at an α = 0.05 are shown by an * in the upper left corner of a panel.

Montreal and Winnipeg exhibit statistically significant declining trends of spatial autocorrelation over the period of study for the middle-income group and, as such, middle-income CTs tend to be adjacent less often over time (Fig 4). In Toronto, the low-income trend is significant and positive, increasing spatial autocorrelation over time. Thus, low-income Toronto CTs tend to be found next to each other more often than chance would predict. That is also true for the medium and high income CTs in Toronto. Conversely, in Montreal and Winnipeg, the negative trend in low-income spatial autocorrelation means that low-income CTs have successively had fewer low-income neighbours over time. The trends in the remaining CMAs are not significant but tend to indicate a positive slope and increasing spatial autocorrelation over time. Except for Calgary, Montreal and Quebec City, there are significant positive trends of spatial autocorrelation for the high-income groups. Neither Ottawa nor Edmonton exhibited any significant spatial autocorrelation in the middle-income class over time. Confidence intervals for trend line slopes of each CMA’s Join-Count data are provided in the supplemental material (S9) in S1 Appendix. The results of Join-Count analyses using the other two neighbourhood configurations yields similar results (S10 and S11 in S1 Appendix).

## Discussion

Prior to the 2010s, there were few studies mapping income polarization in Canada. This research examined the trends in low-, middle- and high-income groups in Canada’s largest CMAs. First and foremost we would like to note that each CMA has its own unique spatial and temporal specificities, whereby differences can be manifested in varying patterns of spatial segregation: unevenness, clustering, exposure, etc [20, 94].

We presented income trends using both the household (Table 1) and individual (Table 2) level incomes. Some researchers prefer to use data on the level of individuals rather than households because they feel that using household data introduces complexities involving changing household size over time [95]. Household size has decreased over time [96], so it is possible that due to the rise in single individual households there might be a decrease in such incomes. Equally possible is that household incomes might increase in some areas in situations where multiple low-income individuals reside together [95]. However, there are all sorts of reasons for decreasing household size, including the fact that households have fewer children now compared to the past. It is therefore important to not generalize suppositions across all time and space.

In a few chapters of the recent Three City Model (TCM) publication [9], it is noted that, per their methodology, Gini coefficients are higher when examining household-income than when examining individual-income. However, earlier research underlines the shortfalls of examining Gini coefficients when examining income inequality and polarization [10]. To avoid overgeneralizing and speculating with the aforementioned structural arguments, we presented trends of low-, middle- and high-income groups in Fig 2 and Tables 1 and 2.

The results between Tables 1 and 2 are in some ways similar. There are statistically significant decreasing trends in the middle-income groups for all but two CMAs (Tables 1 and 2; Fig 2). Almost every CMAs’ income structure is consistent with various conceptualizations of polarization, whose defining feature is the disappearance of the middle whereby the two poles (low and high) increase [40, 93].

The results for household and individual income data are somewhat similar. Income based data is usually more extreme, which suggests that the household-based income data is in most cases a more conservative means of assessing income polarization. Results for Vancouver and Quebec City tend to differ from the other CMAs. For example, no income-group trends are significant in Quebec City for individual-based income data. The converse is the situation with Vancouver, where middle and high-income trends are not significant when household-based income data is examined. As such, there are some differences induced by different income measures.

The low-income groups for all CMAs, except for Quebec City, exhibited significant increasing trend. Of the pairs of datasets where both types of data were statistically significant, in every CMA except for Ottawa-Gatineau, the individual-based income data showed larger increases.

Middle-income group trends were decreasing in every CMA, though Quebec City’s results for individual-based income and Vancouver’s results for household-based income were not statistically significant. Once again, apart from Ottawa-Gatineau, individual based data gave higher extremes than household-based data when comparing how the middle-income groups decreased.

High-income groups give somewhat different results when comparing household- and individual-based income approaches. The household income results which show only half the CMA’s having statistically significant increasing slopes, whereas the individual income approach shows all CMAs except for Ottawa-Gatineau and Quebec City having statistically significant increasing slopes.

The middle-income group has not been equally divided between the high- and low-income groups and there is a clear asymmetry. With few exceptions, the low-income groups have increased significantly more than high-income groups. Such phenomenon is distinctly different from classic polarization, which assumes an equal division of the middle to the high and low ends, and our observations could be termed “lumpenization” (opposite of embourgeoisement).

Historically, it is far easier for one to move from middle-income status downwards, in part because the upper-class is exclusionary. Aside from the trend lines, within the bandwidth curves themselves (Fig 2), the general plateauing of the low-income groups from 2006 onwards and slight increases in the middle-income groups at the same time, may be an indication of stabilization. Patterns of slow or no erosion of the middle-class would be expected in the 1970s, and to an extent the 1980s, as these were a period in which Keynesnian economic policies fostered a strong welfare state [97–99]. However, our results are different, in that they show a steady erosion of the middle-income group during this period. This observation could be due to natural short-term variability of the available census data.

With a shorter record, our trends and inferences would have been different. Our time series was constrained by data availability from 1971 onwards and this is a relatively short period. As such, we are unable to assess any periodicity that might be present in the bandwidth curves themselves and make the assumption that the variation from census to census is random around the trends we produced. As with any time series, and in particular short ones, taking any random interval or start/end point will lead to trends that are in opposition to the full series trend.

We chose the start point of this study as 1971 because the 1970s ushered in a new epoch of re-emerging neighbourhood inequality in Canadian cities [100, 101]. Therefore, we would expect lower levels of change were the start-point of our analysis moved up to a later date.

The high-income bandwidth curves (Fig 2) exhibit a decline around 2001, in almost every CMA. However, the high-income group also exhibits the most variability over the period of record and, similarly to the observations on the middle- and low-income groups, such a decline might simply be a spurious observation resulting from natural periodicity that exists at a greater temporal scale than our observed record. Periodicity is conjecture as we have no way of knowing without a longer time series. Thus, the reliance on the global trend is a more robust way to interpret income polarization over time—as opposed to examining changes in the bandwidth curve direction between individual census years and attempting to explain each ‘kink’. The ‘kinks’ are due to some political/social/local process and we cannot conclusively say that the variability through the period of record is just random variation, it could very well be labor market responses to national and/or provincial policies, changes of national, provincial and municipal governments or any combination thereof plus random variability. Unfortunately, that depth of analysis is beyond the scope of the current research but could prove fruitful in more specific research that aims to understand the observed variability from census year to census year.

We used three bandwidths and analyzed the trend of the average. For clarity, only the middle-income bandwidth (20%) was mapped for all the cities, but similar trends of an expanding low-income area, as depicted in red on our maps (Fig 3 and S6 in S1 Appendix), can be observed irrespective of the range used to define middle-income. These maps were not produced, but the household density equalizing cartograms show similar patterns under other bandwidths of the middle-income group.

The spatial distribution of the low-income area (Fig 3) leads to the inference that suburban areas are increasingly becoming low-income areas. Such spatial patterning is consistent with research which has shown that between 1986 and 2006, poverty and impoverished places have increasingly shifted from the inner-city towards suburbs of large Canadian CMAs [102, 103].

**Fig 5:**
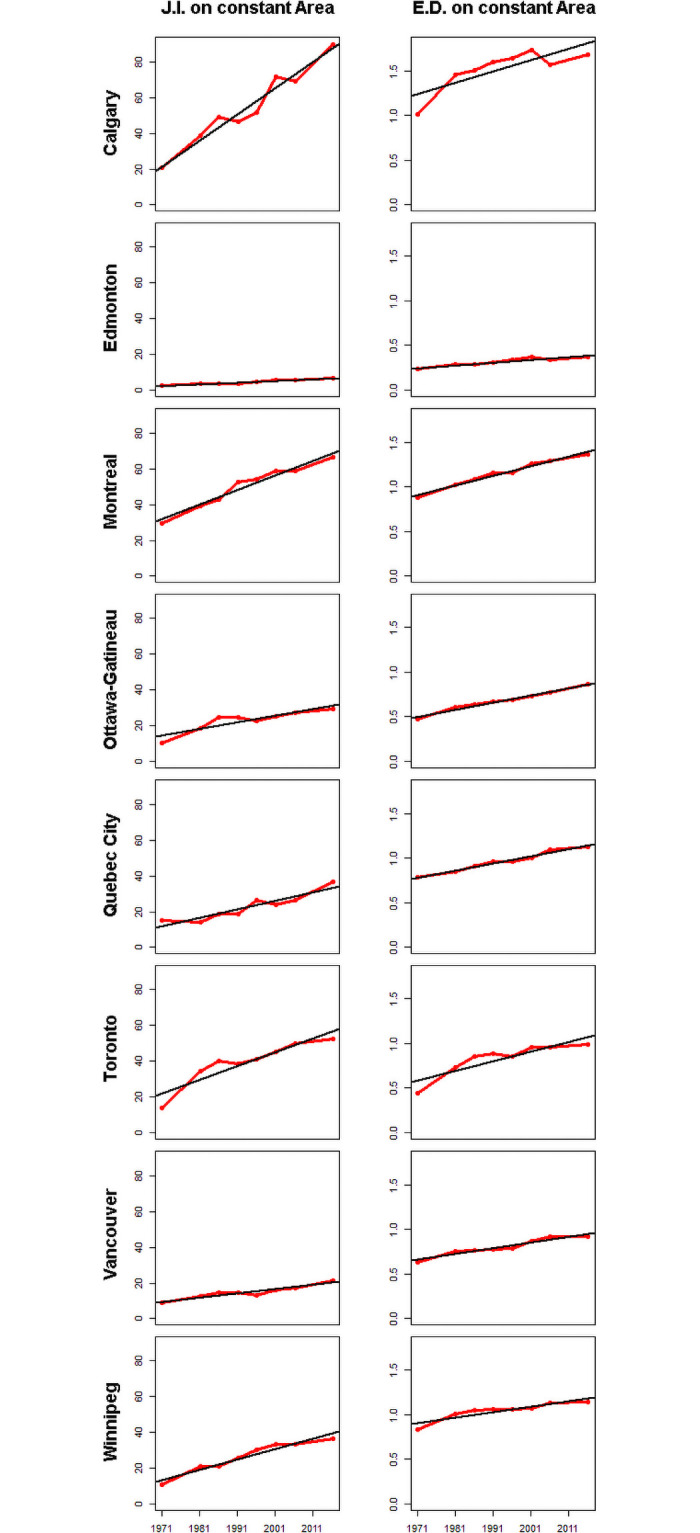
Diagrams showing fragments and fragmentation indexes for Canada’s eight largest CMAs. The first and second columns show results from two fragmentation indices on a constant area over time—the Johnsson fragmentation index (F.I. on constant area) and Edge Density (E.D. on a constant area). Johnsson Fragmentation Index values were multiplied by a million. Confidence intervals from bootstrapping are available in the supplementary material (S8) in S1 Appendix. Every panel in this figure has significant trends at an α = 0.05.

CMAs are increasingly divided, creating a landscape mosaic of concentrated disadvantaged and advantaged groups that we infer from the increasing trends in patch fragmentation over time in all CMAs (Figs 3 and 5). Every CMA has become more fragmented (Fig 5, last two columns). Increasing fragmentation over time and the division of space that ensues is a defining feature of postmodern urban landscapes [104].

The increasing fragmentation of income groups has implications because socioeconomic class remains an important factor in one’s development and opportunities in life. Opportunities in employment, education, health and access to amenities, including health, food, recreational areas and services, are structured by inequalities in a divided urban landscape. For example, affluent parts of cities have more resources and hence opportunities than disadvantages areas [105–109]. The juxtaposition of concentrated advantaged groups and an expanded area of disadvantaged groups produces negative prospects for social cohesion and may contribute to an increased potential of detrimental social backlash.

While the fragmentation indices inform as to what is happening at the spatial scale of the patches that are created by the distribution of high-, medium- and low-income groups in the CMA mosaics, the spatial autocorrelation analysis deals with the behavior of the constituent census tracts (CTs) that create the patches within the urban landscape. The changing nature of spatial autocorrelation among the constituent CTs over time can help to reveal potential spatial processes/interactions that have led to the patch formations through the changes in income status of individual CTs over time. Over time, changes in positive spatial autocorrelation can inform as to the nature of spatial interaction between income-group CTs. For example, do certain income-group CTs attract (cluster), repulse (disperse) or show no change in interaction over time?

With respect to the spatial behavior of individual income groups, the statistically significantly increasing positive spatial autocorrelation trends in all but three of the CMAs for the high-income group equates to a form of spatial interaction whereby, in most CMAs, the high-income CTs tends to attract more high income CTs to form their patches over time. The remaining three CMAs also exhibit positive trends in the spatial autocorrelation that, while non-significant, does reinforce the observation. The growth of high-income patches is due to a spatial process of clustering of high-income CTs. Processes underlying this can be many, for example, gentrification in general or spillover gentrification effects in particular, would be local processes that can lead to this type of clustering among high-income CTs. Other processes include professionalization [110], a process in which individuals become upwardly socially mobile through the attainment of professional skills.

Gentrification is a socio-spatial process that is operating in every CMA among the inner-city CTs around downtown cores (Fig 5), and this is well documented in past research [111–119]. Maps of inner-city areas and their creation are provided in the appendix (S12 in S1 Appendix). Coincident with gentrification of the downtown core, many CTs will show increases in average household incomes. Such change reflects a more affluent population creating emerging clusters of wealth which replace previous populations that had lower or middle incomes.

The low-income CTs exhibited, for the most part, trends of positive spatial autocorrelation that increased over time, but these were not statistically significant. While the total area within the CMAs that is occupied by the low-income group is growing, that growth may be more opportunistic: middle-income CTs are scavenged wherever they occur. However, the positive trends over time are suggestive of weaker process whereby some low-income CTs are more likely to end up near low-income CTs to form patches. An additional potential factor that contributes to this result is a compositional effect, as there is an increase over time of one- and two-person income households [20, 94]. However, the rise of one and two person households is not spatially equal or homogeneous and hence one should be wary of generalizing this observation across all temporal and spatial domains.

The middle-income group CTs in Winnipeg and Montreal show a significant decreasing trend in spatial autocorrelation consistent with CTs where the space around them is dissimilar with respect to income-groups over time. That is consistent with dispersion of middle-income CTs within the landscape mosaic over time as well as fragmentation at the CT level and patch level. The decreasing magnitude of positive spatial autocorrelation could be due to the disappearing middle-income groups in these CMAs. However, six of the eight CMAs have no significant trends in the spatial autocorrelation of the middle-income groups over time. While fragmentation is increasing at the patch level, the behavior of middle-income CTs clustering with other middle-income CTs has not changed over time and this is likely due to the fact that this group has been disappearing from all CMA mosaics. Perhaps, from a spatial perspective, that is a weakness: middle-income CTs are not increasingly attracting other middle-income CTs to their patches and so are more vulnerable to ‘predation’.

Perhaps the lack of statistically significant positive or negative trends in the spatial autocorrelation among middle- and low-income CTs, makes these more vulnerable to being appropriated by the CTs of the high-income group that shows increasing clustering over time. In short, the CMAs are becoming more fragmented and only the high-income group CTs exhibit increasing spatial organization in the form of clustering over time. The high-income group CTs are the most spatially organized and in some sense this group is in ‘control’ of the mosaic. Similar research has found that urban areas all have their own unique spatial and temporal specificities, whereby differences can be manifested in varying patterns of spatial segregation: unevenness, clustering, exposure, etc [20, 94].

The underlying explanation that has produced the income polarization and emergent fragmented spatial mosaics is multifaceted and includes neoliberal economic policies (which work towards abating the welfare state), globalization, and immigration. Delving deeply into these components is beyond the scope of our study and evidence, but in the following paragraphs it is worth conjecturing how these mechanisms could potentially influence the patterns we have found.

The dismantling of the Canadian welfare state can be seen through the dismantling of various benefits, and these vary both among provinces and within provinces [48, 120]. Additionally, the 1980s and 1990s saw the polarization of the labor market [121], and consequently a reduction of the population in the middle-income category is to be expected.

Immigration plays a role in the evolving income structure of the city and is the product of two concurrent forces. The first is that suburban regions are increasingly becoming reception zones of immigrants as the inner-city becomes less affordable [122], whereas the second is an unfortunate pattern whereby incomes of recent immigrants, in relation to that of native born Canadians, have been significantly decreasing since the 1980s [123–125]. In Anglo-America, immigrant reception areas are increasingly in suburban areas more so than the inner-city [126–128], and coincidentally many suburban areas are comprised of low-income CTs.

In the US, the urban structure is extremely divided (often along racial lines) [57, 70, 129–133]. A dated but popular argument made by Goldberg and Mercer [134] suggests that Canadian cities are ostensibly distinct from their American counterparts. However, as this paper demonstrates empirical evidence of the rising income polarization in Canadian cities as well as how that trend is leading to a more spatially divided urban mosaic that resembles American counterparts.

## Conclusion

Canada’s fortuity in terms of egalitarianism is uncertain. As noted, amongst OECD countries, Canada has seen the highest increase of income inequality in recent years [3, 45, 48–52]. Inequality is exemplified at the urban scale, where we illustrate a disappearing middle-income populace both spatially and temporally that is expressed by an increasingly spatially fragmented urban income mosaic. This study has demonstrated the unequivocal decreases of middle-income groups over time and coincident increases in the low-income groups in the eight largest CMAs in Canada.

This research finds significant growth in low-income areas within every CMA examined, many of which are located in declining areas that are often referred to as inner-suburbs. Concurrently, levels of fragmentation have increased universally with only the high-income groups exhibiting any increase in spatial organization through the process of spatial clustering of similar CTs. This study provides a base for a more thorough examination of the lived experiences of individuals in these increasingly divided Canadian urban landscape mosaics.

Finally, our research has identified places in CMAs where policy makers can focus on, the actions of which may lead to the fostering of middle-income communities and the confrontation of what some have termed the “menace of suburban decline” [135]. Such endeavor may mitigate or reverse the decline of middle-income groups in metropolitan areas. Whereas this research does not postulate remedies to inequality and polarization, it should be noted that in order to successfully combat the aforementioned problems, a multi-pronged approach is required [125].

## Supporting information

S1 Appendix (DOCX)
