# Correction: The temporal evolution of income polarization in Canada’s largest CMAs

**DOI:** 10.1371/journal.pone.0315962

**Published:** 2024-12-12

**Authors:** Lazar Ilic, M. Sawada

The authors made erroneous calculations in 10 cells of Table 2 regarding 1971 for Montreal and Winnipeg. As a result, interpolated data for 1976 was wrong and the final bootstrapped results has been affected. In addition, a data transcription error was made for Vancouver, whereby the low- and high-income data were swapped. Finally, Calgary’s medium- & high-income group was adjusted by a very small amount due to a data transcriptional mistake. Please see the correct Table 2 here.

**Table 2:** Slopes of the three income groups (low, middle & high) and their associated 95% confidence intervals (CI) obtained via non-parametric bootstrapping of individual income data.

CMA	Low (95% CI)	CI_mid_	CI_high_
Calgary	0.57 [0.45, 0.74]*	-0.65 [-0.86, -0.52]*	0.09 [0.05, 0.13]*
Edmonton	0.35 [0.27, 0.43]*	-0.48 [-0.60, -0.32]*	0.13 [0.05, 0.18]*
Montreal	0.14 [0.10, 0.18]*	-0.20 [-0.26, -0.14]*	0.06 [0.04, 0.09]*
Ottawa-Gatineau	0.08 [0.05, 0.15]*	-0.12 [-0.23, -0.04]*	0.05 [-0.02, 0.12]
Quebec City	0.02 [-0.02, 0.06]	-0.05 [-0.14, 0.02]	0.03 [-0.01, 0.8]
Toronto	0.53 [0.41, 0.66]*	-0.62 [-0.76, -0.48]*	0.09 [0.07, 0.13]*
Vancouver	0.40 [0.35, 0.48]*	-0.51 [-0.61, -0.43]*	0.11 [0.08, 0.16]*
Winnipeg	0.19 [0.05, 0.29]*	-0.32 [-0.50, -0.09]*	0.13 [0.05, 0.24]*

To reflect the updated Table 2, the correct fifth and sixth sentence of the first paragraph of Results are: Three-quarters of the high-income group trends are significant if we consider individual income (Table 2). In general, middle and low-income groups show similar trends for both types of income, household or individual. The greatest variability is around the low and middle-income groups.

The correct fifth paragraph of Discussion is: The results for household and individual income data are somewhat similar. Individual income-based data is sometimes more extreme, which suggests that the household-based income data is in some cases a more conservative means of assessing income polarization. Results for Vancouver and Quebec City tend to differ from the other CMAs. For example, no income-group trends are significant in Quebec City for individual-based income data. The converse is the situation with Vancouver, where middle- and high-income trends are not significant when household-based income data is examined. As such, there are some differences induced by different income measures.

The correct sixth paragraph of Discussion is: The low-income groups for all CMAs, except for Quebec City, exhibited significant Increasing trends. Of the pairs of datasets where both types of data were statistically significant, in CMAs other than Montreal, Ottawa-Gatineau, and Winnipeg, the individual-based income data showed larger increases than the household-based income data.

The correct second sentence of seventh paragraph of Discussion is: Once again, apart from Montreal, Ottawa-Gatineau, and Winnipeg individual-based data gave higher extremes than household-based data when comparing how the middle-income groups decreased.
