# Supplementary material for: The temporal evolution of income polarization in Canada’s largest CMAs
Source: PLoS One. 2021 Jun 8;16(6):e0251430. doi: 10.1371/journal.pone.0251430 (PMC8186789; doi:10.1371/journal.pone.0251430)

**The Temporal Evolution of Income Polarization in Canada’s Largest CMAs**

Authors: Ilic, Lazar & Sawada, M.
Laboratory for Applied Geomatics and GIS Science (LAGGISS), Department of Geography, Environment and Geomatics, University of Ottawa, Ottawa, Canada

**Contents**

**S1. Three City Project Traction
S2. Population ranges of CTs in CMAs.**

**S3. Comparison of four geographic scales**

**S4. Full census variable names**

**S5. Shapefile errors and corrections**

**S6. Fragmentation Index tests on Spatial scales**

**S7. Maps of CMAs**

**S8. Confidence intervals of bootstrapped Fragmentation Index data**

**S9. Confidence intervals of bootstrapped Joins-Count data (Queens spatial weight matrix)**

**S10. Trends of Joins-Count data and Confidence intervals of bootstrapped Joins-Count data (Rooks spatial weight matrix)**

**S11. Trends of Joins-Count data and Confidence intervals of bootstrapped Joins-Count data (KNN-5 weight matrix)**

**S12. Inner City Extents**

**S1. Three City Project Traction**

To examine traction of the Three City Project, data was tabulated per individual study per year. The data source was Google Scholar, and was obtained on August 1, 2019.

|  | **2007** | **2008** | **2009** | **2010** | **2011** | **2012** | **2013** | **2014** | **2015** | **2016** | **2017** | **2018** | **2007-2018** |
| --- | --- | --- | --- | --- | --- | --- | --- | --- | --- | --- | --- | --- | --- |
| **Toronto** | 0 | 10 | 16 | 14 | 26 | 28 | 44 | 46 | 75 | 43 | 67 | 60 | 403 |
| **Vancouver** |  |  |  |  |  |  | 1 | 5 | 10 | 8 | 5 | 8 | 37 |
| **Montreal** |  |  |  |  |  |  |  | 2 | 2 | 2 |  | 3 | 9 |
| **Halifax** |  |  |  |  |  |  |  | 1 | 5 | 2 | 3 | 2 | 13 |
| **Hamilton** |  |  |  |  |  |  |  |  | 1 | 4 | 6 | 6 | 17 |
| **Calgary** |  |  |  |  |  |  |  |  |  |  |  | 1 | 1 |
| **Total:** | 0 | 10 | 16 | 14 | 26 | 28 | 45 | 54 | 93 | 59 | 81 | 80 |  |

The search criteria for each city was simple the name of each study. Bellow are the search criteria and the links which they yielded. Once the link was obtained, the “custom range” option allowed for searching for total citation numbers in individual years.

Toronto: “The three cities within Toronto”

https://scholar.google.com/scholar?cites=17836097183110024094&as_sdt=2005&sciodt=0,5&hl=en

Vancouver: “Divisions and disparities in lotus-land: Socio-spatial income polarization in greater Vancouver, 1970-2005”

https://scholar.google.com/scholar?client=firefox-b-d&um=1&ie=UTF-8&lr&cites=799435445609479635

Montreal: “A City-Region Growing Apart? Taking Stock of Income Disparity in Greater Montréal, 1970-2005”

https://scholar.google.com/scholar?sxsrf=ACYBGNSNpR4u0t8Wp8fOCauWaC2MIlwY_w:1579500673657&uact=5&um=1&ie=UTF-8&lr&cites=9157432325877169501

Hamilton: “A city on the cusp: Neighbourhood change in Hamilton since 1970”

https://scholar.google.com/scholar?client=firefox-b-d&um=1&ie=UTF-8&lr&cites=14588784678578239141

Halifax : “Neighbourhood Change in Halifax Regional Municipality,. 1970 to 2010: Applying the “Three Cities” Model”

https://scholar.google.com/scholar?client=firefox-b-d&um=1&ie=UTF-8&lr&cites=9530011185545756078

Calgary: “Socio-Spatial Polarization in an Age of Income Inequality: An Exploration of Neighbourhood Change in Calgary’s “Three Cities””

https://scholar.google.com/scholar?client=firefox-b-d&sxsrf=ACYBGNTd_Pm7830yZPAoXasf7egA_57X_A:1579500866522&uact=5&um=1&ie=UTF-8&lr&cites=3745678424521638426

**S2. Population ranges of CTs in CMAs.**

a. Calgary

| Year | Total CTs w Pop | Low | High | Tracts wPop 2500 to 8000 | Perc of Total |
| --- | --- | --- | --- | --- | --- |
| 1971 | 77 | 985 | 11290 | 73 | 94.805 |
| 1981 | 115 | 1342 | 14987 | 104 | 90.435 |
| 1986 | 143 | 2117 | 9546 | 133 | 93.007 |
| 1991 | 153 | 2129 | 9160 | 144 | 94.118 |
| 1996 | 153 | 2091 | 17323 | 132 | 86.275 |
| 2001 | 193 | 617 | 11798 | 176 | 91.192 |
| 2006 | 202 | 308 | 20946 | 173 | 85.644 |
| 2016 | 253 | 1196 | 23798 | 218 | 86.166 |

b. Edmonton

| Year | Total CTs w Pop | Low | High | Tracts wPop 2500 to 8000 | Perc of Total |
| --- | --- | --- | --- | --- | --- |
| 1971 | 88 | 155 | 25735 | 68 | 77.273 |
| 1981 | 142 | 2 | 9907 | 121 | 85.211 |
| 1986 | 180 | 41 | 9119 | 156 | 86.667 |
| 1991 | 186 | 69 | 10424 | 166 | 89.247 |
| 1996 | 187 | 45 | 12078 | 158 | 84.492 |
| 2001 | 204 | 41 | 13390 | 177 | 86.765 |
| 2006 | 219 | 43 | 21628 | 171 | 78.082 |
| 2016 | 268 | 5 | 28192 | 204 | 76.119 |

c. Montreal

| Year | Total CTs w Pop | Low | High | Tracts wPop 2500 to 8000 | Perc of Total |
| --- | --- | --- | --- | --- | --- |
| 1971 | 570 | 45 | 20515 | 414 | 72.632 |
| 1981 | 661 | 4 | 14584 | 478 | 72.315 |
| 1986 | 709 | 122 | 13275 | 528 | 74.471 |
| 1991 | 738 | 120 | 15180 | 527 | 71.409 |
| 1996 | 757 | 261 | 20845 | 532 | 70.277 |
| 2001 | 850 | 160 | 11422 | 664 | 78.118 |
| 2006 | 864 | 246 | 11912 | 674 | 78.009 |
| 2016 | 959 | 5 | 20786 | 744 | 77.581 |

d. Ottawa-Gatineau

| Year | Total CTs w Pop | Low | High | Tracts wPop 2500 to 8000 | Perc of Total |
| --- | --- | --- | --- | --- | --- |
| 1971 | 120 | 95 | 20240 | 90 | 75.000 |
| 1981 | 178 | 25 | 7598 | 151 | 84.831 |
| 1986 | 191 | 45 | 10084 | 162 | 84.817 |
| 1991 | 208 | 56 | 10468 | 176 | 84.615 |
| 1996 | 215 | 45 | 12210 | 172 | 80.000 |
| 2001 | 236 | 50 | 16103 | 201 | 85.169 |
| 2006 | 248 | 574 | 12049 | 204 | 82.258 |
| 2016 | 277 | 5 | 16747 | 221 | 79.783 |

e. Quebec City

| Year | Total CTs w Pop | Low | High | Tracts wPop 2500 to 8000 | Perc of Total |
| --- | --- | --- | --- | --- | --- |
| 1971 | 98 | 60 | 14680 | 56 | 57.143 |
| 1981 | 126 | 154 | 12695 | 89 | 70.635 |
| 1986 | 138 | 300 | 11182 | 103 | 74.638 |
| 1991 | 152 | 284 | 10080 | 122 | 80.263 |
| 1996 | 152 | 240 | 12369 | 115 | 75.658 |
| 2001 | 165 | 230 | 7888 | 132 | 80.000 |
| 2006 | 165 | 225 | 9686 | 132 | 80.000 |
| 2016 | 181 | 61 | 9935 | 133 | 73.481 |

f. Toronto

| Year | Total CTs w Pop | Low | High | Tracts wPop 2500 to 8000 | Perc of Total |
| --- | --- | --- | --- | --- | --- |
| 1971 | 447 | 35 | 20120 | 290 | 64.877 |
| 1981 | 606 | 11 | 20583 | 494 | 81.518 |
| 1986 | 726 | 47 | 14864 | 629 | 86.639 |
| 1991 | 808 | 59 | 14213 | 700 | 86.634 |
| 1996 | 808 | 58 | 25437 | 675 | 83.540 |
| 2001 | 929 | 47 | 20635 | 820 | 88.267 |
| 2006 | 998 | 51 | 22724 | 886 | 88.778 |
| 2016 | 1150 | 10 | 23401 | 975 | 84.783 |

g. Vancouver

| Year | Total CTs w Pop | Low | High | Tracts wPop 2500 to 8000 | Perc of Total |
| --- | --- | --- | --- | --- | --- |
| 1971 | 179 | 75 | 24475 | 118 | 65.922 |
| 1981 | 245 | 560 | 10513 | 212 | 86.531 |
| 1986 | 273 | 137 | 13317 | 239 | 87.546 |
| 1991 | 298 | 162 | 12215 | 261 | 87.584 |
| 1996 | 298 | 168 | 19736 | 237 | 79.530 |
| 2001 | 386 | 155 | 11914 | 334 | 86.528 |
| 2006 | 409 | 145 | 13367 | 357 | 87.286 |
| 2016 | 474 | 10 | 13855 | 403 | 85.021 |

h. Winnipeg.

| Year | Total CTs w Pop | Low | High | Tracts wPop 2500 to 8000 | Perc of Total |
| --- | --- | --- | --- | --- | --- |
| 1971 | 106 | 90 | 12320 | 79 | 74.528 |
| 1981 | 135 | 9 | 13779 | 104 | 77.037 |
| 1986 | 147 | 457 | 11191 | 121 | 82.313 |
| 1991 | 155 | 410 | 7907 | 129 | 83.226 |
| 1996 | 157 | 387 | 10049 | 124 | 78.981 |
| 2001 | 164 | 373 | 8329 | 135 | 82.317 |
| 2006 | 167 | 231 | 9739 | 135 | 80.838 |
| 2016 | 173 | 228 | 20465 | 140 | 80.925 |

**S3. Comparison of four geographic scales**
Appearance of search result counts for terms from select journals.
Many of these journals are in the top ten impact factor journals for urban studies related research. Additional journals in the list are ones in which much urban research is published, but which are not specifically limited to urban themes.

| **Journal Name** | **Census Tract** | **Block Group** | **Dissemination Area** | **Enumeration Area** |
| --- | --- | --- | --- | --- |
| Annals of the American Association of Geographers | 156 | 51 | 3 | 8 |
| Canadian Geographer | 90 | 4 | 16 | 17 |
| City | 2 | 0 | 0 | 0 |
| Cities | 121 | 27 | 5 | 4 |
| International Journal of Urban and Regional Research | 42 | 4 | 1 | 2 |
| Journal of Urban Economics | 278 | 51 | 0 | 2 |
| Professional Geography | 147 | 42 | 1 | 5 |
| Regional Science and Urban Economics | 173 | 47 | 1 | 2 |
| Urban Geography | 262 | 55 | 3 | 6 |
| Urban Studies | 315 | 76 | 5 | 4 |
| Urban Affairs Review | 144 | 34 | 0 | 0 |

**S4. Full census variable names**

1971 Census :

-Households: Income from all sources: under $3,000

-Households: Income from all sources: under $3,000-$4,999

-Households: Income from all sources: under $5,000-$6,999

-Households: Income from all sources: under $7,000-$9,999

-Households: Income from all sources: under $10,000-$14,999

-Households: Income from all sources: under $15,000 & over

-Households: Average income from all sources

1981 Census :

-Private household income - all households

-Private household income - all households - average income

1986 Census :

-Household income - all private households

-Average income (note: from category, "Household income - all private households")

1991 Census :

-Household income - all private households

-Average income, household income $ (note: from category, "Household income - all private households")

1996 Census :

-Household income of private households (20% sample data)

-Average household income $ (note: from category, "Household income of private households (20% sample data)")

2001 Census :

-Household income in 2000 of private households - 20% sample data

-Average household income $ (note: from category, "Household income in 2000 of private households - 20% sample data")

2006 Census :

-Household income in 2005 of private households - 20% sample data

-Average household income $ (note: from category, "Household income in 2005 of private households - 20% sample data")

2016 Census :

- Total – Income statistics in 2015 for private households by household size – 25% sample data

-Average total income of households in 2015 ($)

Sources:

1971, 1981, 1986, 1991, 1996, 2001, 2006: Canadian Census Analyzer database

2016 : Statistics Canada IVT table: “98-401-X2016043” Census Profile - Age, Sex, Type of Dwelling, Families, Households, Marital Status, Language, Income, Immigration and Ethnocultural Diversity, Housing, Aboriginal Peoples, Education, Labour, Journey to Work, Mobility and Migration, and Language of Work for Census Metropolitan Areas, Tracted Census Agglomerations and Census Tracts, 2016 Census

**S5. Shapefile errors and corrections**

Shapefiles were created by Statistics Canada and are available on the Scholars Geoportal Database, located at http://geo2.scholarsportal.info . The following CMAs in the select years had errors which were corrects.

**Montreal 1971.**

Tract 852 – This CT had two polygons. One part was mislabeled. Fixed by merging them.

**Montreal 1981.**

Tract 411 – This CT was mislabeled as CT 418. Fixed by renaming.
 Tract 560 – There were two CTs with this name. Fixed by merging.

Tract 584 – This CT was missing. It had to be redrawn.

Tract 756.01 – There were two polygons with this name. Fixed by merging.

Tract 825.01 – This CT was mislabeled as tract 852.01... Fixed by renamed.

Tract 886 – There were two polygons with this name. Fixed by merging.

Tract 887.01 – There were two polygons with this name. Fixed by merging.

Tract 887.02 – This CT was mislabeled as CT 887.01. Fixed by renaming.

Tract 904 – There were two polygons with this name. Fixed by merging.

**Ottawa 1981.**

Tract 151.02 – contained two tracts by mistake. Fixed using the explode tool and

subsequent renaming of new polygons into CTs 151.02 and 151.03.

**Vancouver 1981.**

Tract 200 – This CT was missing. Fixed by using explode tool and subsequent renaming

of new polygons into CTs 200 and 202.

Tract 237 – This CT was missing or merged into CT 243. Fixed by redrawing.

Tract 141 – This CT was mislabeled. There were two CTs labeled 140. Fixed by

renaming. Tract 141 is further west than tract 140.

Tract 285.01 – This CT was mislabeled as CT 235.01. Fixed by renaming.

**Toronto 1981.**

Tract 165 – This CT was mislabeled as CT 166. Fixed by renaming.

Tract 203 – This CT was mislabeled as CT 208. Fixed by renaming.

Tract 516.08 – This CT mislabeled as CT 516.07. Fixed by renaming.

**S6**. **Fragmentation Index tests on Spatial scales**

The two following cases were examined to test the Johnson Fragmentation Index and Edge Density. Both examples prove inadequate in situations where the spatial scale is very different.

Case A:
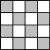
 Case B:
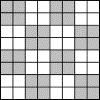


Johnson’s Fragmentation Index gives a result of 1 for Case A (15 / 15) and 0.238 for Case B (15 / 63).

Edge Density gives a result of 4 for Case A (64 / 16) and 2 for Case B (128 / 64).

The two cases should have the same results as visually they depict the exact same pattern.

**S7. Maps**

Higher resolution maps are available at the following github page: https://github.com/lazarification

a. Calgary


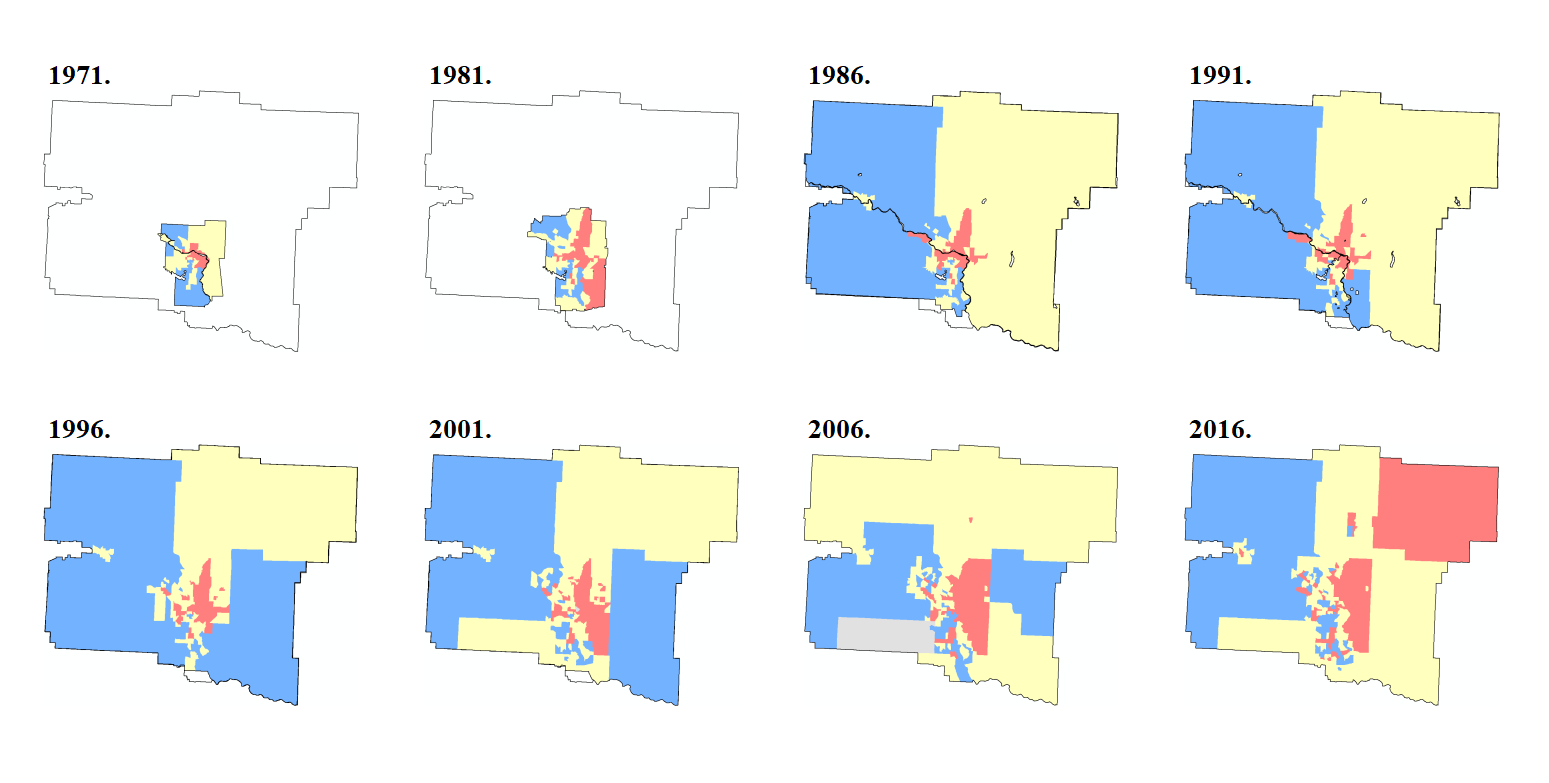


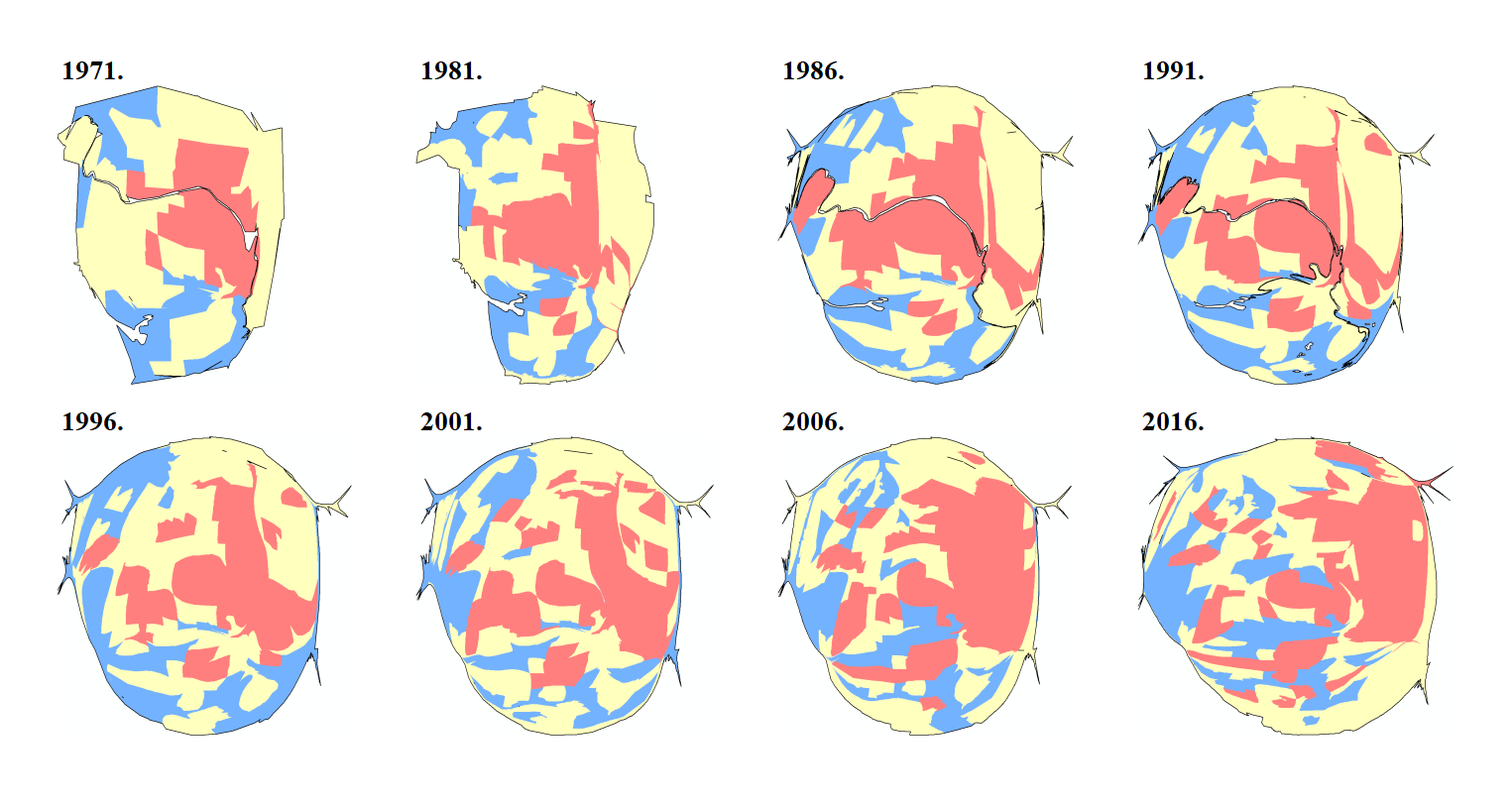


b. Edmonton


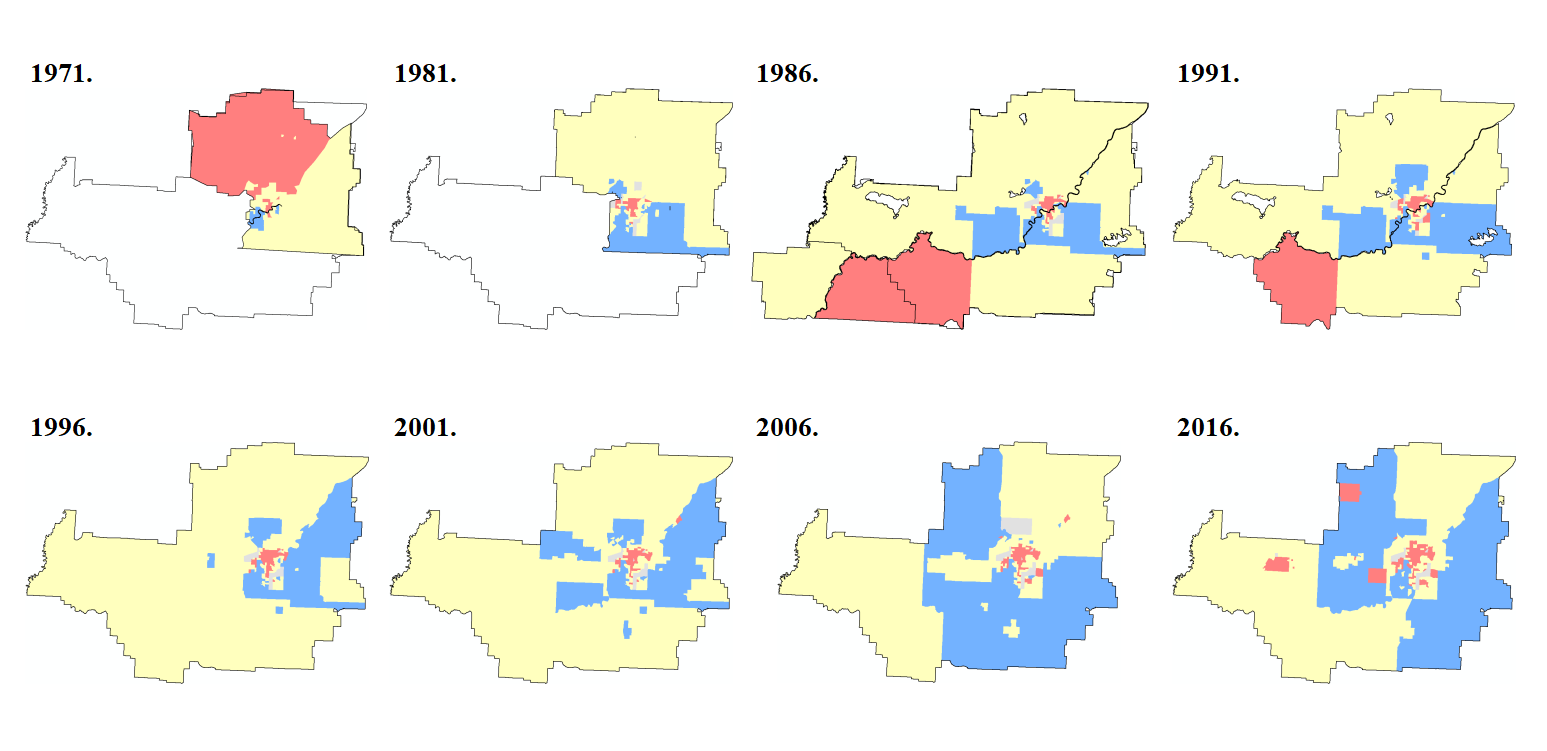


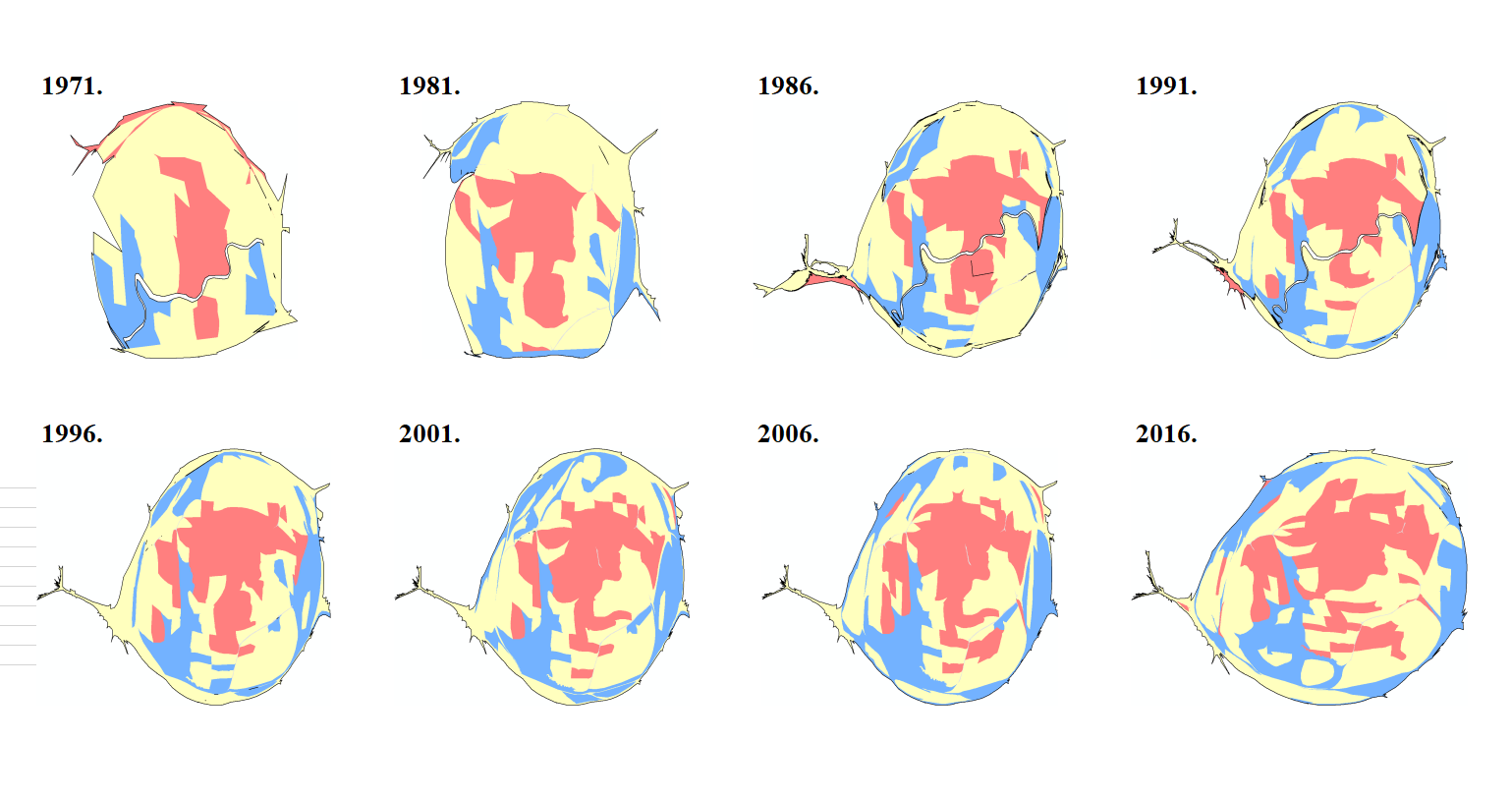


c. Montreal


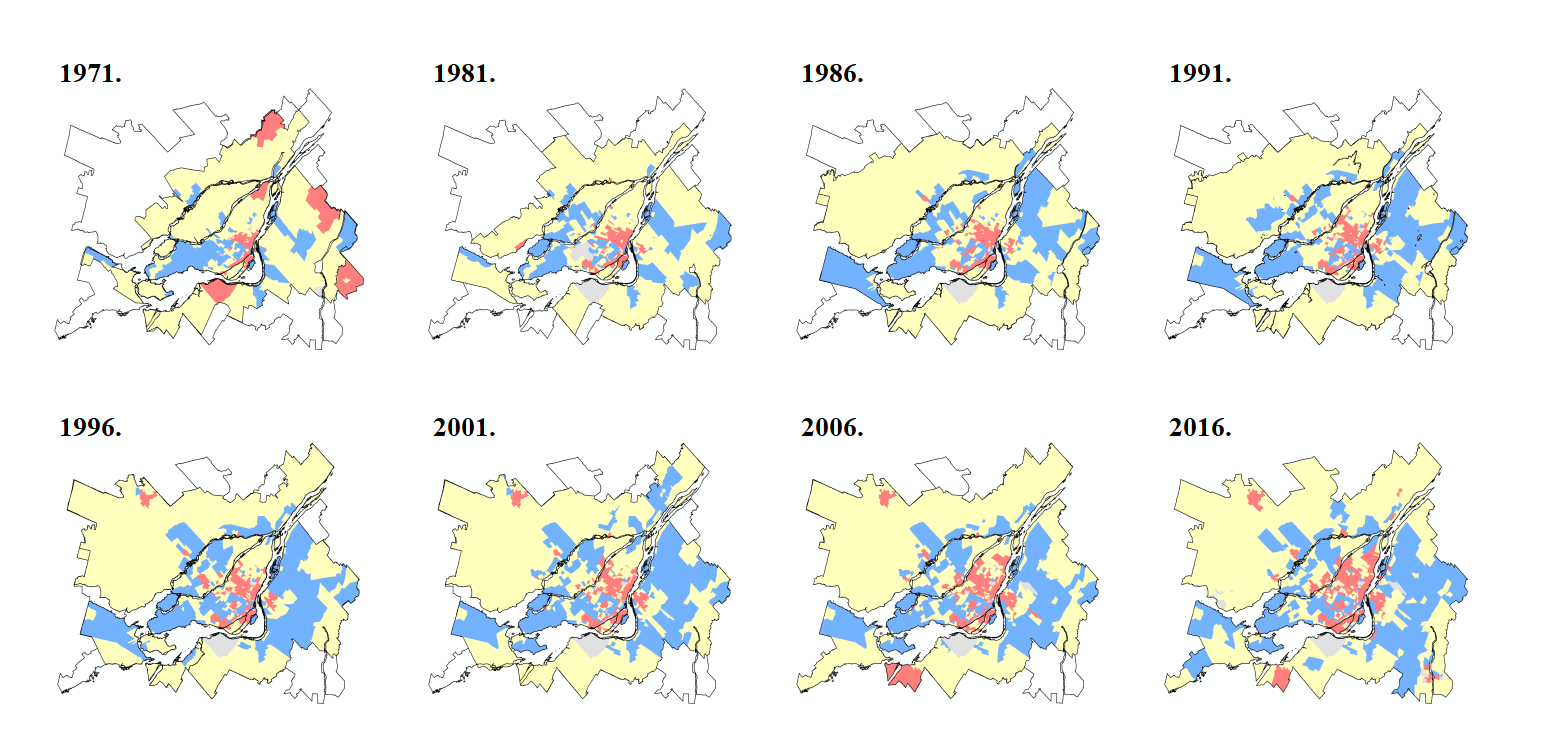


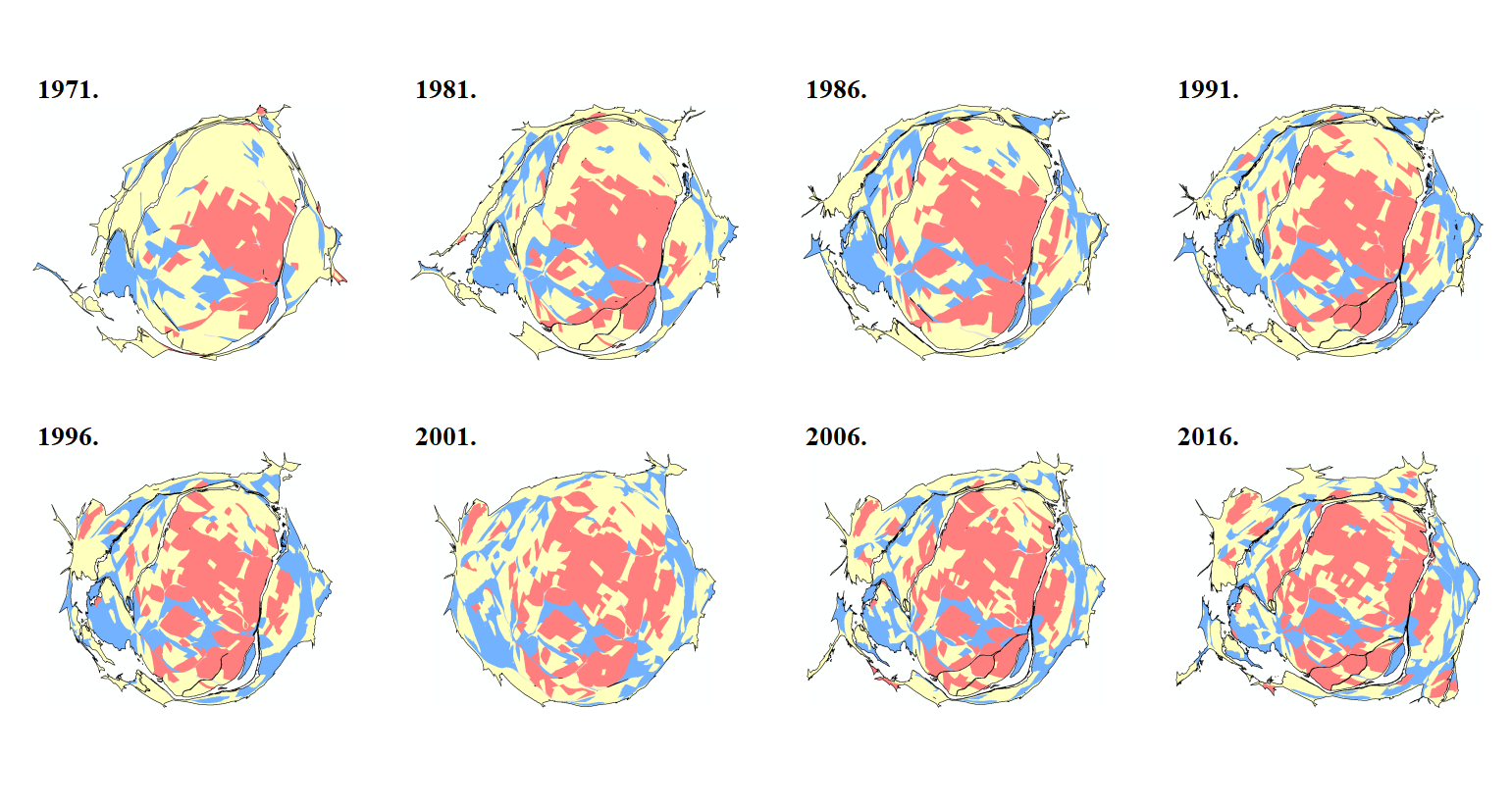


d. Ottawa-Gatineau


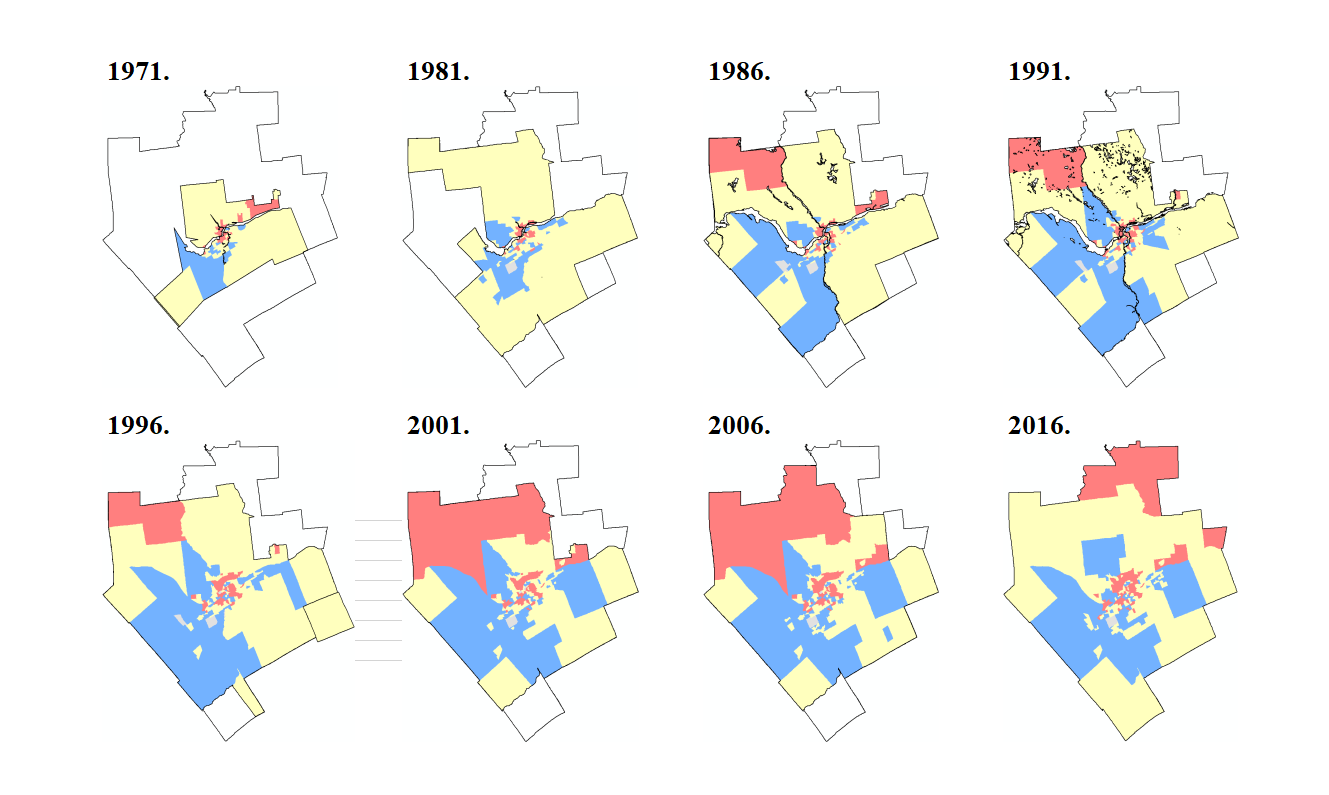


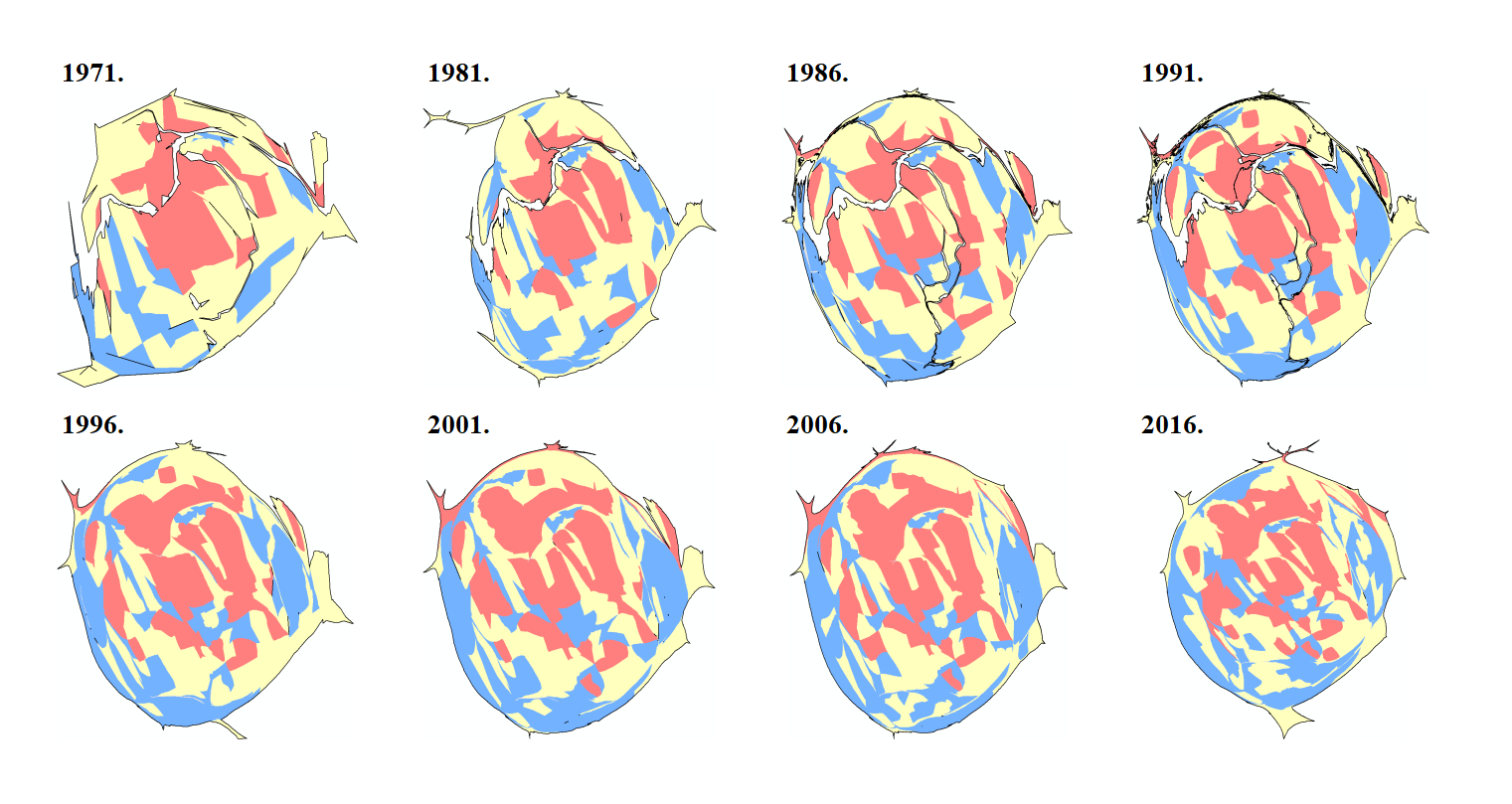


e. Quebec City


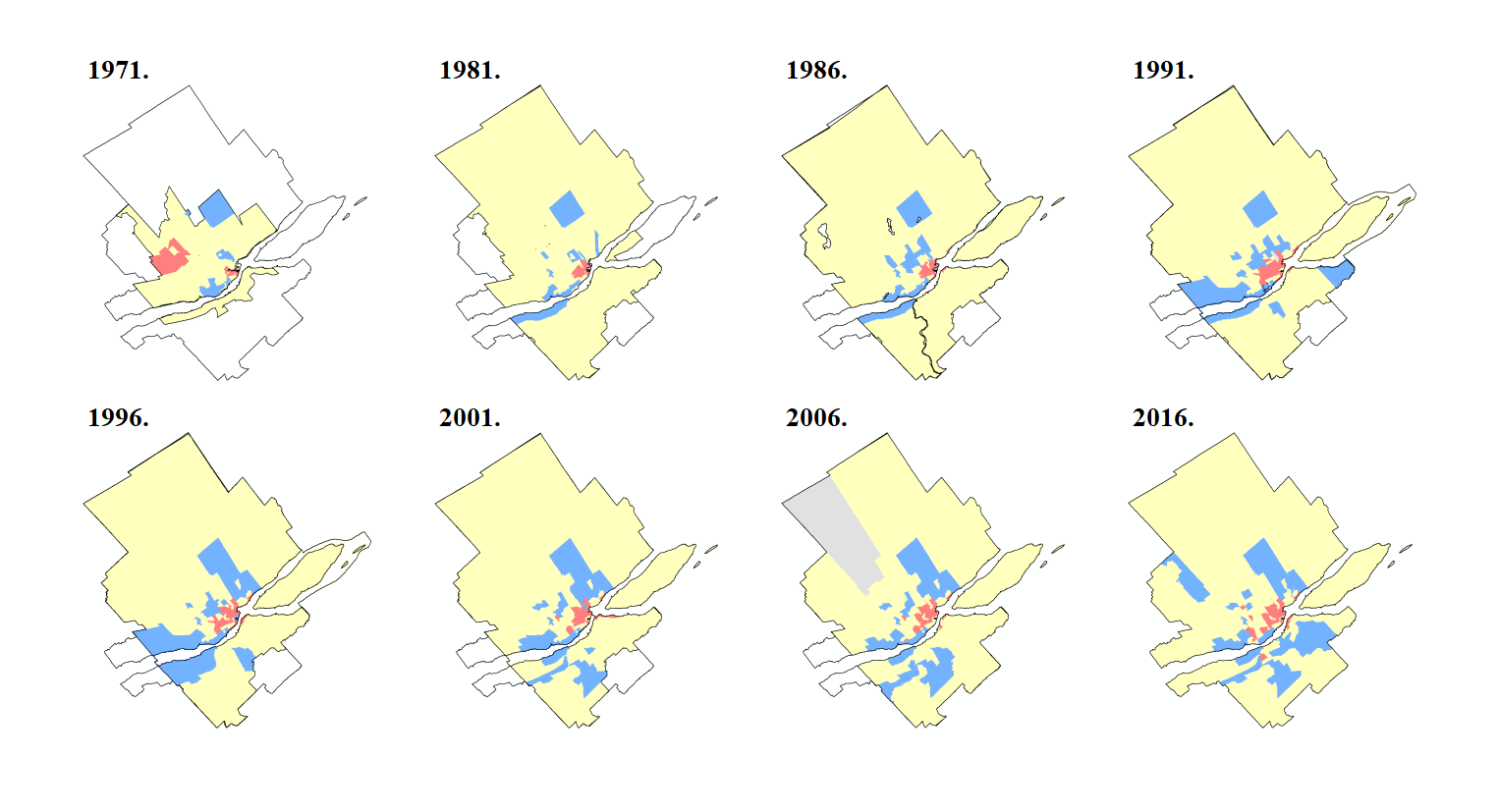


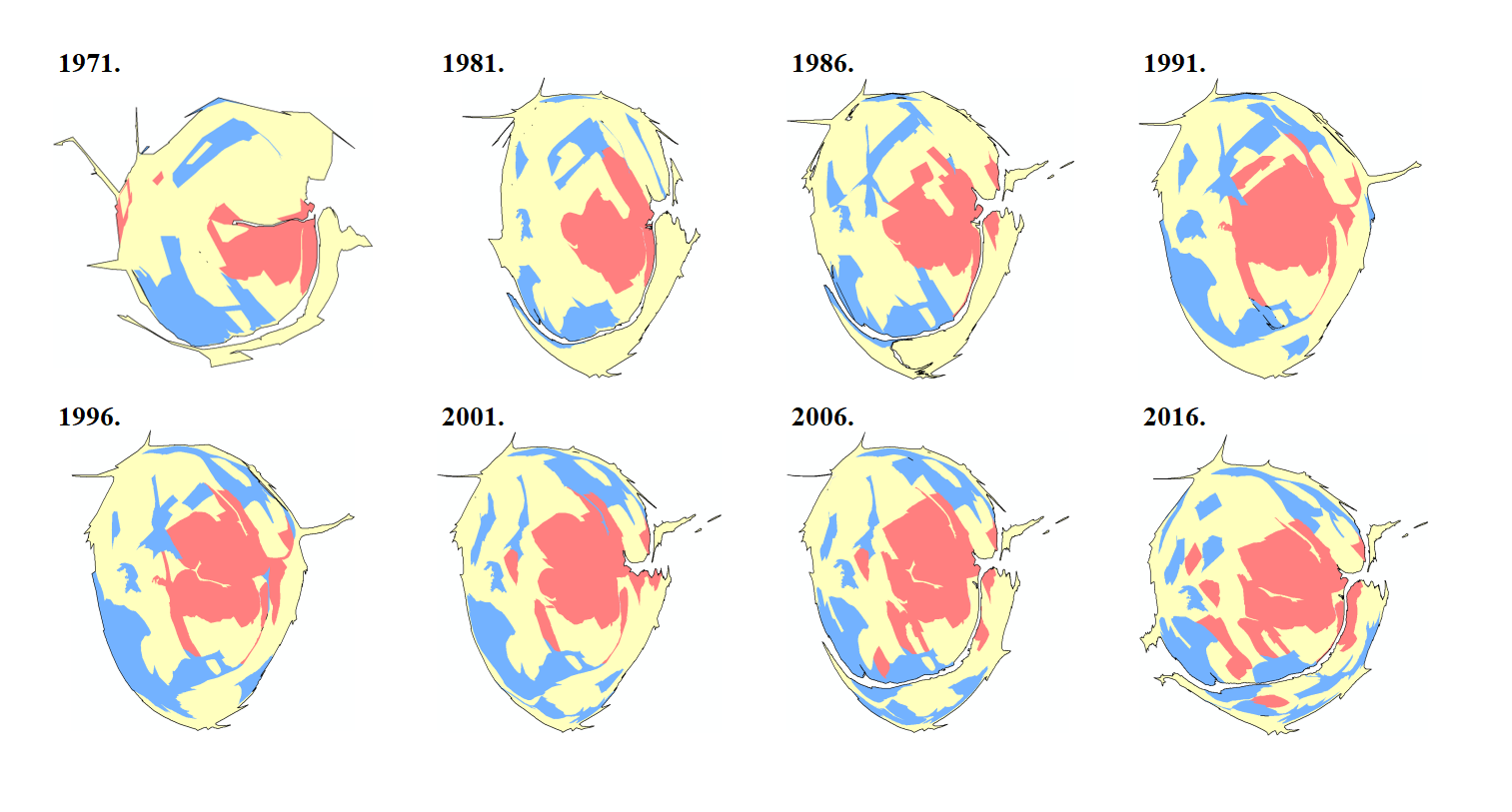


f. Toronto

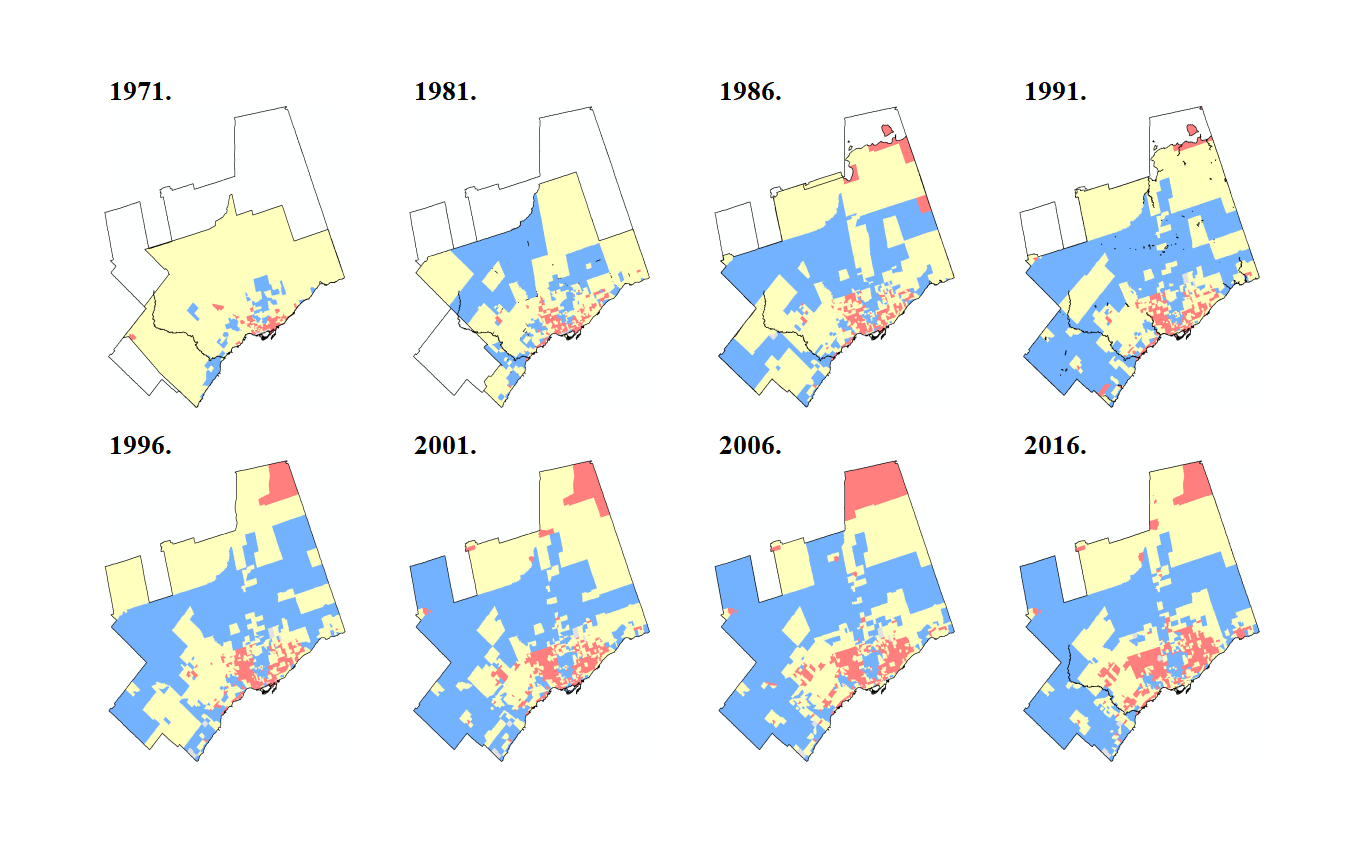


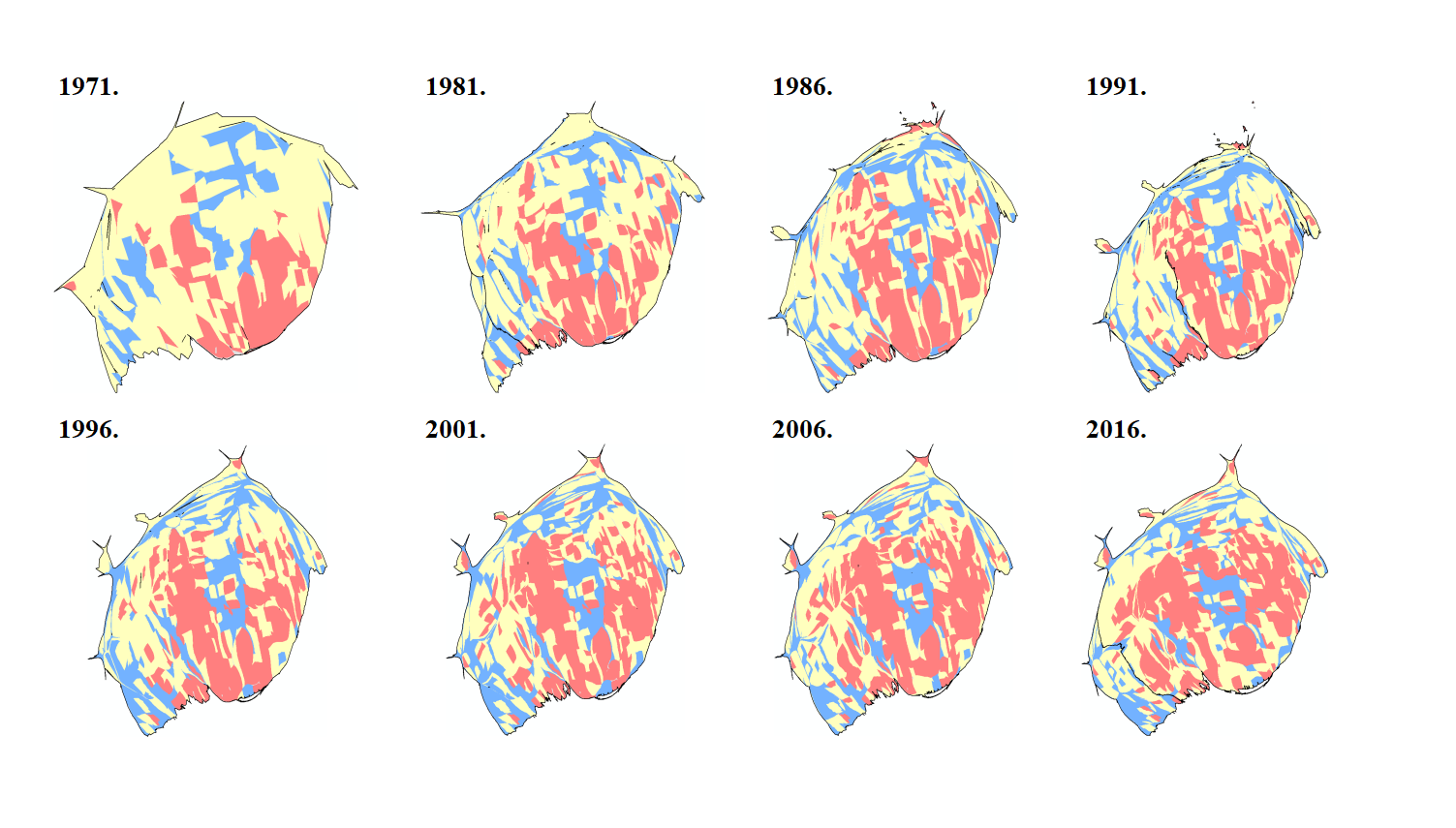


g. Vancouver


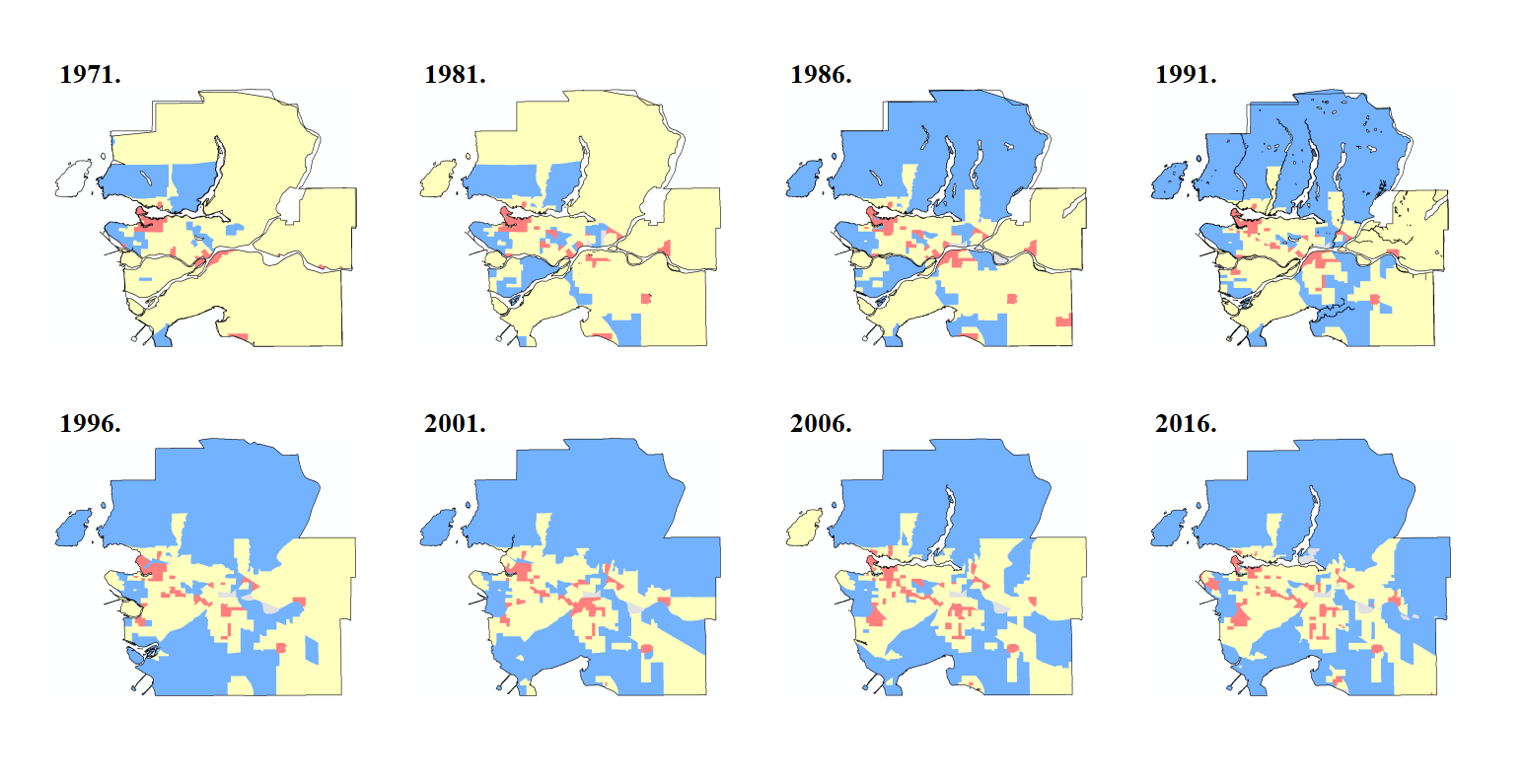


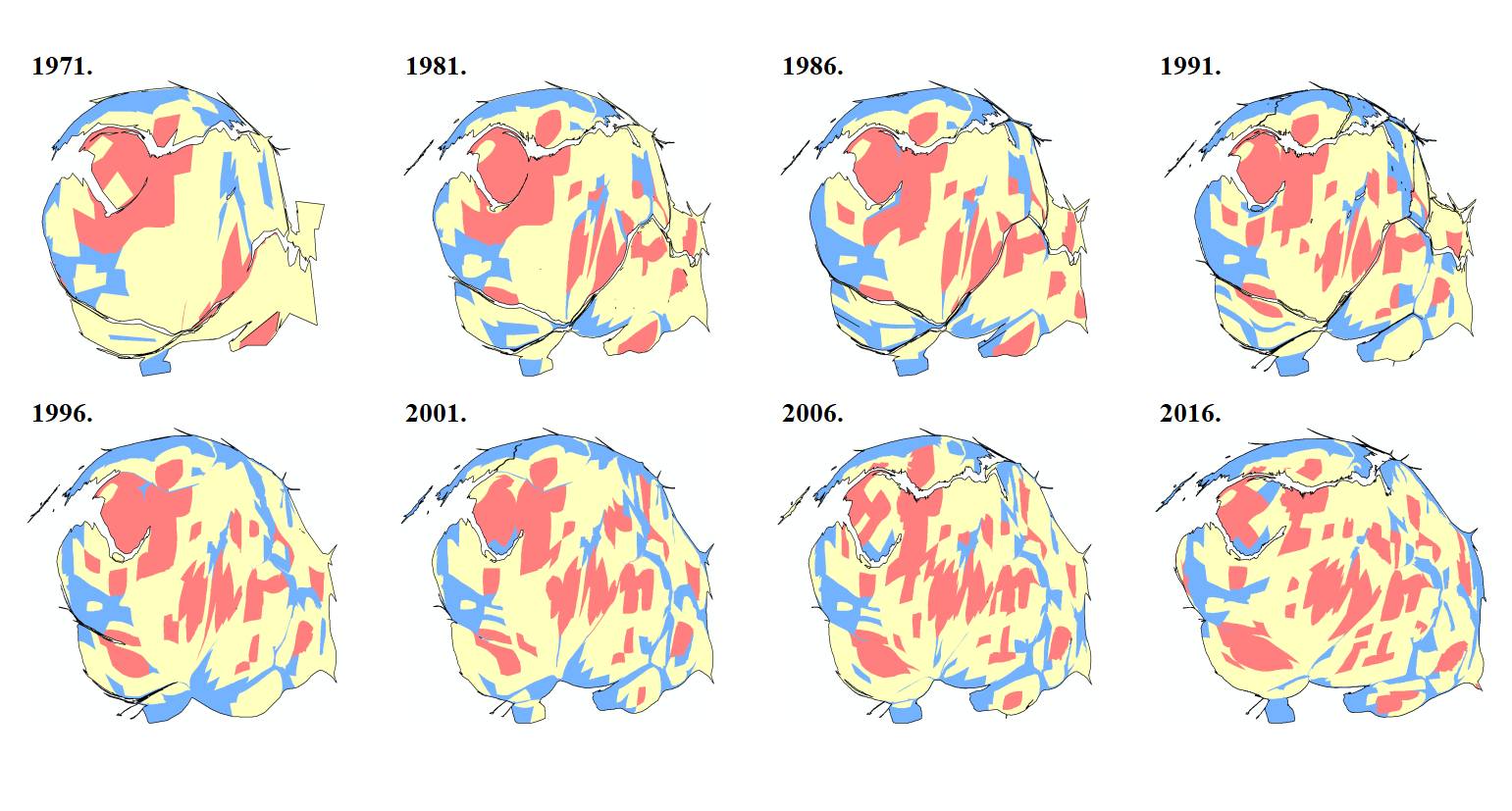


h. Winnipeg

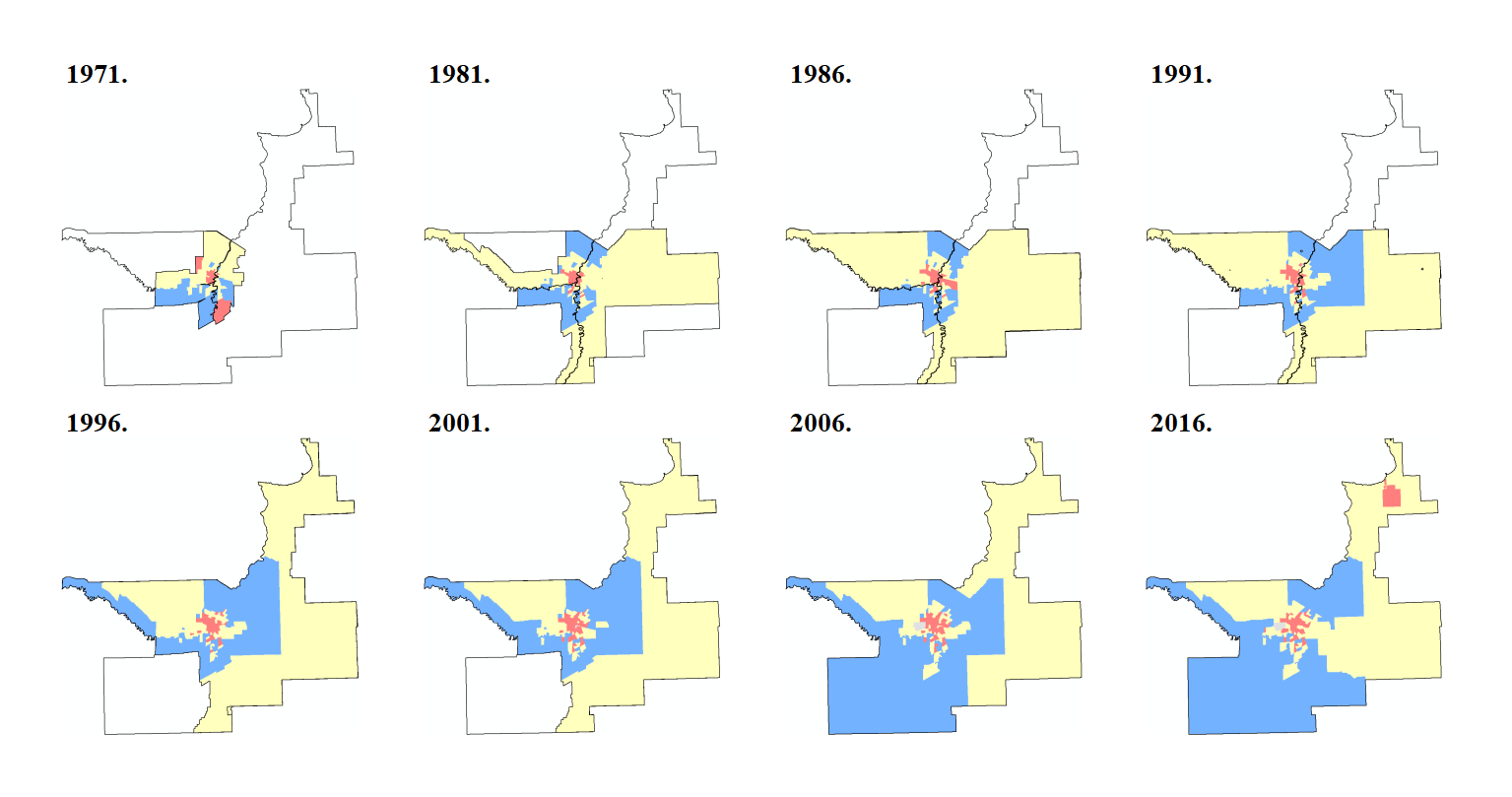


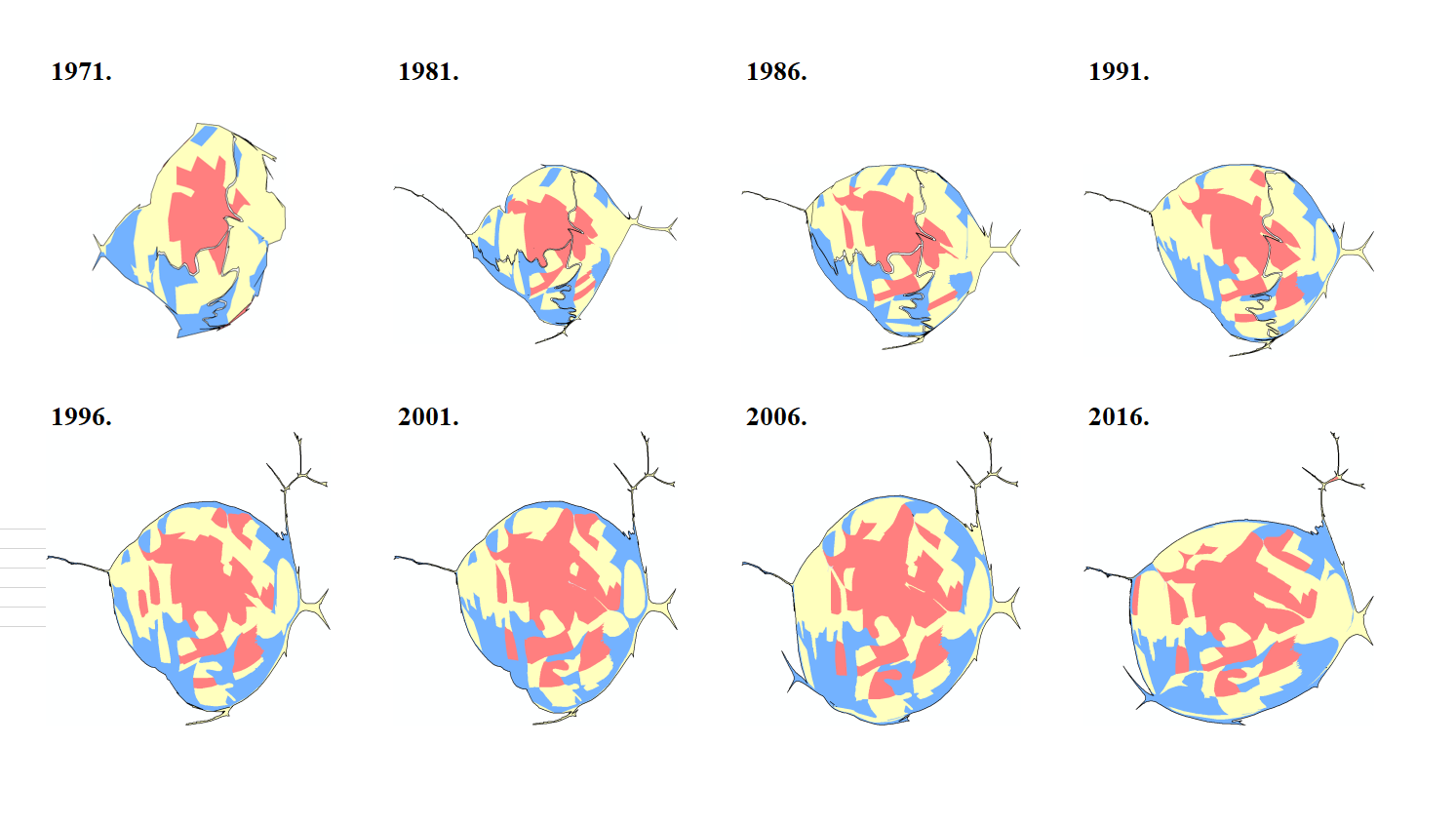


**S8. Confidence intervals of bootstrapped Fragmentation Index data**

Slopes of two Fragmentation Indices (Johnsson Index and Edge Density) and their associated 95% confidence intervals (CI) obtained via non-parametric bootstrapping. All slopes are statistically significant.

| **CMA** | **JI (95% CI)** | **CI_ED_** |
| --- | --- | --- |
| Calgary | 1.47 [1.14, 1.80] | 0.013 [0.003, 0.022] |
| Edmonton | 0.10 [0.07, 0.12] | 0.003 [0.002, 0.004] |
| Montreal | 0.82 [0.65, 0.99] | 0.011 [0.009, 0.012] |
| Ottawa-Gatineau | 0.37 [0.19, 0.56] | 0.008 [0.007, 0.009] |
| Quebec City | 0.48 [0.29, 0.67] | 0.008 [0.007, 0.009] |
| Toronto | 0.77 [0.44, 1.10] | 0.011 [0.005, 0.016] |
| Vancouver | 0.24 [0.15, 0.33] | 0.006 [0.004, 0.008] |
| Winnipeg | 0.57 [0.42, 0.73] | 0.006 [0.003, 0.009] |

**S9. Confidence intervals of bootstrapped Joins-Count data**

Slopes of the three groups (Low, Middle & High) and their associated 95% confidence intervals (CI) obtained via non-parametric bootstrapping. A slope is significant statistically when its confidence interval does not include zero and these are indicated by an *.

| **CMA** | **Low (95% CI)** | **CI_mid_** | **CI_high_** |
| --- | --- | --- | --- |
| Calgary | 0.03 [-0.02, 0.07] | 0.04 [-0.00, 0.08] | 0.04 [-0.02, 0.09] |
| Edmonton | 0.02 [-0.05, 0.08] | 0.03 [-0.02, 0.09] | 0.18 [0.13, 0.22]* |
| Montreal | -0.05 [-0.09, -0.00]* | -0.07 [-0.12, -0.03]* | 0.06 [-0.00, 0.12] |
| Ottawa-Gatineau | 0.04 [-0.01, 0.09] | 0.00 [-0.02, 0.02] | 0.06 [0.03, 0.09]* |
| Quebec City | 0.03 [-0.04, 0.10] | 0.01 [-0.03, 0.04] | 0.04 [-0.02, 0.09] |
| Toronto | 0.16 [0.10, 0.22]* | 0.04 [-0.04, 0.11] | 0.27 [0.20, 0.35]* |
| Vancouver | 0.01 [-0.03, 0.05] | -0.03 [-0.07, 0.00] | 0.15 [0.11, 0.19]* |
| Winnipeg | -0.02 [-0.06, 0.01] | -0.03 [-0.06, -0.01]* | 0.12 [0.07, 0.16]* |

**S10. Trends of Joins-Count data and Confidence intervals of bootstrapped Joins-Count data (Rooks spatial weight matrix)**

| **CMA** | **Low (95% CI)** | **CI_mid_** | **CI_high_** |
| --- | --- | --- | --- |
| Calgary | 0.03 [-0.02, 0.07] | 0.04 [-0.00, 0.08] | 0.04 [-0.02, 0.09] |
| Edmonton | 0.02 [-0.05, 0.08] | 0.03 [-0.02, 0.09] | 0.18 [0.13, 0.22]* |
| Montreal | -0.05 [-0.09, -0.00]* | -0.07 [-0.12, -0.03]* | 0.06 [-0.00, 0.12] |
| Ottawa-Gatineau | 0.04 [-0.01, 0.09] | 0.00 [-0.02, 0.02] | 0.06 [0.03, 0.09]* |
| Quebec City | 0.03 [-0.04, 0.10] | 0.01 [-0.03, 0.04] | 0.04 [-0.02, 0.09] |
| Toronto | 0.16 [0.10, 0.22]* | 0.04 [-0.04, 0.11] | 0.27 [0.20, 0.35]* |
| Vancouver | 0.01 [-0.03, 0.05] | -0.03 [-0.07, 0.00] | 0.15 [0.11, 0.19]* |
| Winnipeg | -0.02 [-0.06, 0.01] | -0.03 [-0.06, -0.01]* | 0.12 [0.07, 0.16]* |


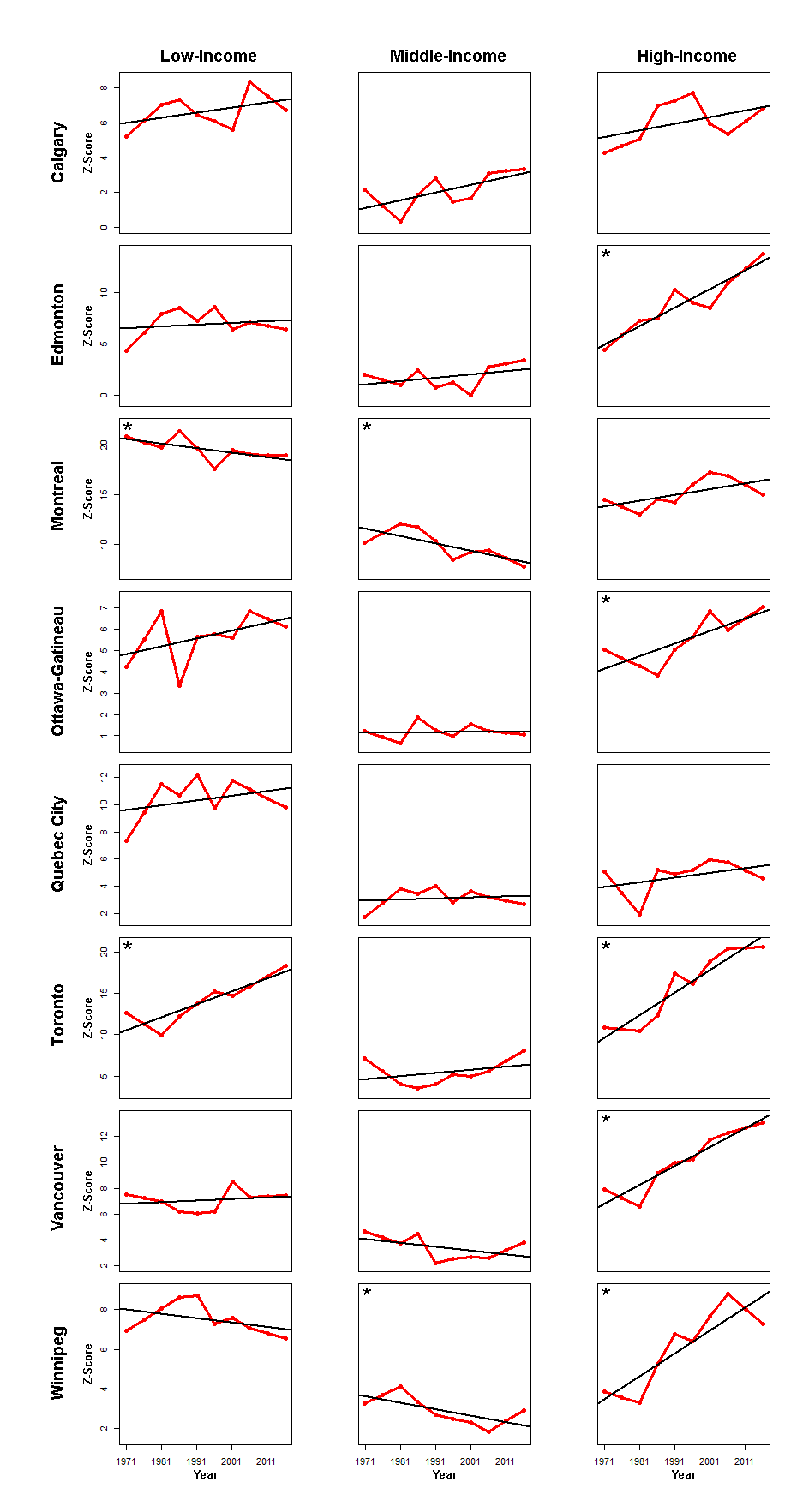


**S11. Trends of Joins-Count data and Confidence intervals of bootstrapped Joins-Count data (KNN-5 spatial weight matrix)**

| **CMA** | **Low (95% CI)** | **CI_mid_** | **CI_high_** |
| --- | --- | --- | --- |
| Calgary | 0.07 [0.00, 0.14] | -0.01 [-0.05, 0.03] | 0.11 [0.06, 0.15]* |
| Edmonton | 0.05 [-0.00, 0.11] | -0.02 [-0.07, 0.04] | 0.05 [-0.02, 0.12] |
| Montreal | -0.05 [-0.09, -0.02]* | -0.15 [-0.19, -0.10]* | 0.02 [-0.05, 0.09] |
| Ottawa-Gatineau | 0.05 [-0.01, 0.10] | 0.01 [-0.01, 0.02] | 0.07 [0.00, 0.14]* |
| Quebec City | 0.03 [-0.05, 0.12] | 0.04 [-0.00, 0.09] | 0.02 [-0.04, 0.08] |
| Toronto | 0.23 [0.15, 0.31]* | 0.09 [0.04, 0.14]* | 0.20 [0.14, 0.25]* |
| Vancouver | 0.04 [-0.02, 0.10] | 0.01 [-0.04, 0.07] | 0.09 [0.05, 0.13]* |
| Winnipeg | -0.05 [-0.07, -0.03]* | -0.01 [-0.03, 0.01] | -0.02 [-0.07, 0.03] |


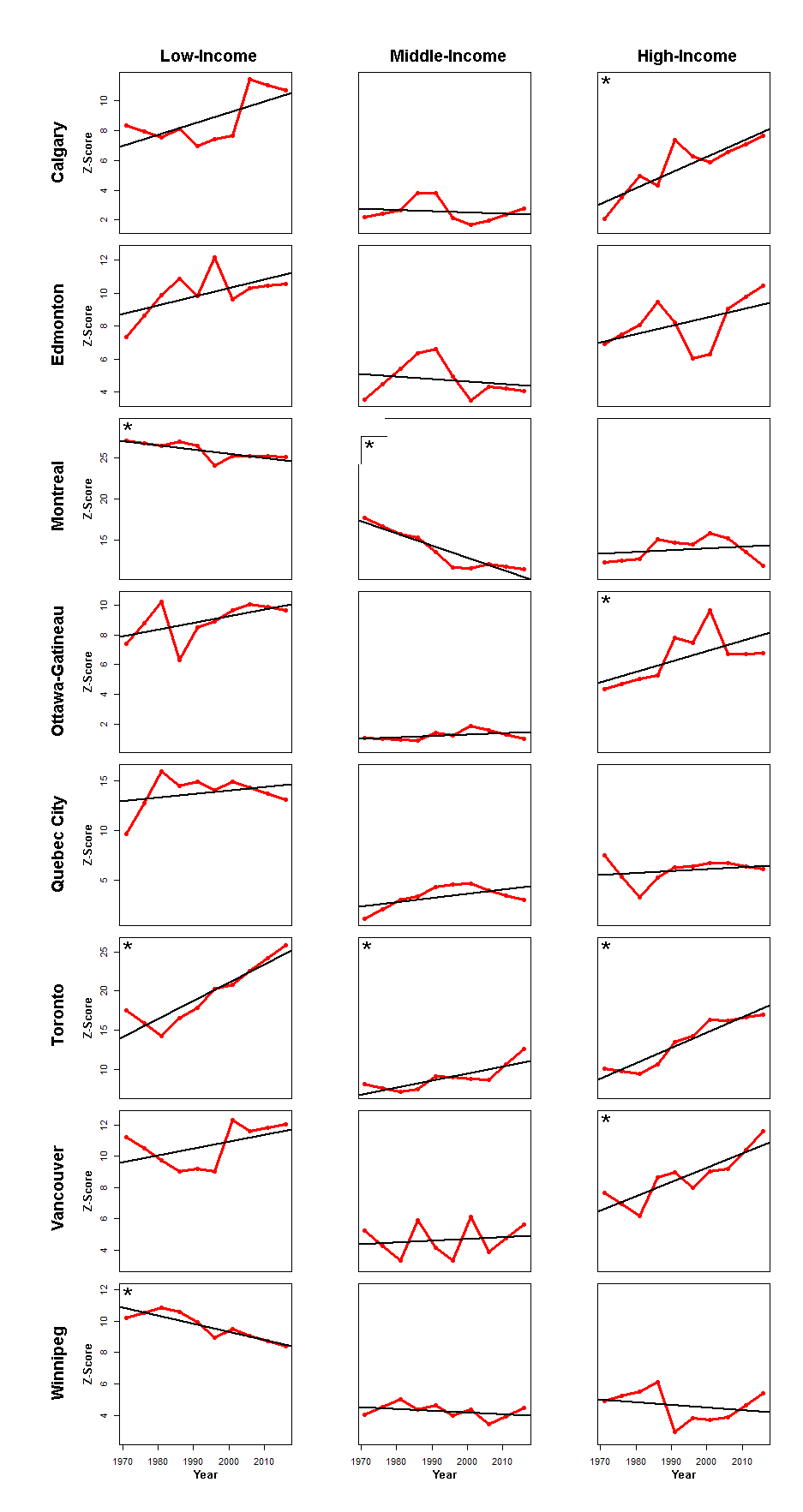


**S12. Inner City Extents**

Numerous studies have analyzed the Canadian inner-city. However, there is not a consensus as the extent of the inner-city. In the Canadian context, the inner-city is an area that is usually defined by having a large percentage of housing stock which was built before a particular date which is usually around World War II.

The census contains data on the age of the housing stock. Hence, one can see how many housing units were built during and prior particular periods of time. Using data at the census tract level, it is possible to discern which areas have an older housing stock that is reflective of the “inner-city”.
 In the present study the goal is not to determine the most precise definition of what constitutes a particular CMA’s “inner-city” region. However, the maps which were produced are based on insights from the following sources:

Bourne, L.S. 1992. Population turnaround in the Canadian inner city: Contextual factors and social consequences, Canadian Journal of Urban

Research, 1, pp. 69-92.

Broadway, Michael. 1992. DIFFERENCES IN INNER‐CITY DEPRIVATION: AN ANALYSIS OF SEVEN CANADIAN CITIES. Canadian

Geographer / Le Géographe canadien, 36: 189-196. doi:10.1111/j.1541-0064.1992.tb01131.x

Broadway, Michael & Jesty, Gillian. 1998. Are Canadian Inner Cities Becoming More Dissimilar? An Analysis of Urban Deprivation Indicators.

Urban Studies, 35(9), 1423–1438. https://doi.org/10.1080/0042098984213

Carter, Tom., Polevychok, Chesya. & Sargent, Kurt. 2003. Is Winnipeg’s Aboriginal Population Ghettoized? Canada Research Chair in Urban

Change and Adaptation. Research Highlight No. 2. Winnipeg: The Institute of Urban Studies, The University of Winnipeg.

City of Calgary. Municipal Development Plan Maps. Retrieved from:

https://www.calgary.ca/_layouts/cocis/DirectDownload.aspx?target=http%3a%2f%2fwww.calgary.ca%2fPDA%2fpd%2fDocuments

%2fmunicipal-development-plan%2fmdp-maps.pdf&noredirect=1&sf=1

Ley, David. 1985. Gentrification in Canadian inner cities: patterns, analysis, impact and policy. Vancouver: University of British Columbia,

Deptartment of Geography.

Ley, David. 1988. SOCIAL UPGRADING IN SIX CANADIAN INNER CITIES. Canadian Geographer / Le Géographe canadien, 32: 31-45.

doi:10.1111/j.1541-0064.1988.tb00853.x

Ley, David. 1992. GENTRIFICATION IN RECESSION: SOCIAL CHANGE IN SIX CANADIAN INNER CITIES, 1981-1986, Urban

Geography, 13:3, 230-256, DOI: 10.2747/0272-3638.13.3.230

Lezubski, Darren & Silver, Jim. 2015. High and Rising Revisited: Changes in Poverty and Related Inner City Characteristics 1996 to 2011. In,

Drawing on Our Strengths: State of the Inner City Report 2015. Winnipeg: Canadian Centre for Policy Alternatives Manitoba Office.

P. 7-34.

Lorch, Brian. 2015. Spatial Polarization of Income in a Slow-Growth City. In, The Divided Prairie City: Income Inequality Among Winnipeg’s

Neighbourhoods, 1970–2010. Eds. Jino Distasio and Andrew Kaufman. Winnipeg: Institute of Urban Studies, The University of

Winnipeg. P. 14-25.

Savard, Charles. 2007. La lutte de la Coalition pour la sauvegarde des écoles des quartiers centraux de Québec (Vieux-Limoilou 2004-2006).

Montréal: Centre de recherche sur les innovations sociales.

Trudi Bunting, Alan R. Walks & Pierre Filion (2004) The uneven geography of housing affordability stress in Canadian metropolitan areas,

Housing Studies, 19:3, 361-393, DOI: 10.1080/0267303042000204287

Villeneuve, P. & Trudelle, C. (2008). Retour au centre à Québec : la renaissance de La Cité est-elle durable ? Recherches sociographiques, 49 (1),

25–45. https://doi.org/10.7202/018192ar

Walks, R. A. (2001). The Social Ecology of the Post-Fordist/Global City? Economic Restructuring and Socio-spatial Polarisation in the Toronto

Urban Region. Urban Studies, 38(3), 407–447. https://doi.org/10.1080/00420980120027438

The aforementioned sources are not in total agreement as to what constitutes the inner-city in certain cities. To an extent this is to be expected, because as decades pass, one might define the inner-city as a broader area than previously. Hence, the inner-city areas that we offer are approximate rather than definite.

Maps of the locations of inner cities within CMAs are provided:

a. Calgary, b. Edmonton, c. Montreal, d. Ottawa-Gatineau,

e. Quebec City, f. Toronto, G. Vancouver, H. Winnipeg


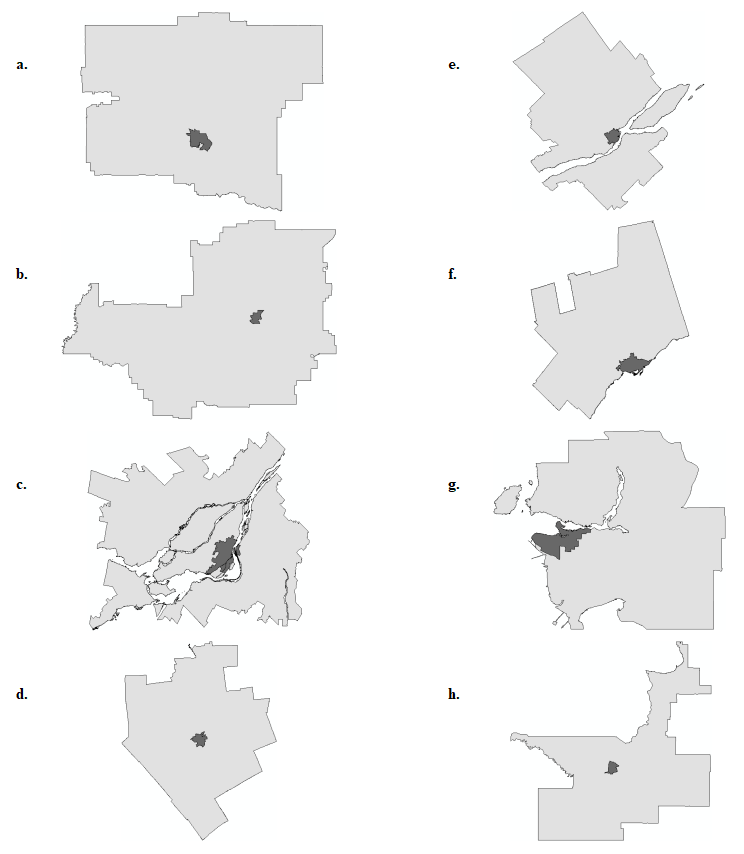

Supplement: S1 Appendix — (DOCX) [file pone.0251430.s001.docx]
